# Enhancing structural to functional brain network prediction using topological graph learning

**DOI:** 10.3389/fnins.2026.1939985

**Published:** 2026-09-28

**Authors:** Shashipraba N. K. Rajakaruna, Asim K. Dey, Snigdhansu Chatterjee

**Affiliations:** 1 Department of Mathematics and Statistics, Texas Tech University, Lubbock, TX, United States; 2 Department of Mathematics and Statistics, University of Maryland, Baltimore County, Baltimore, MA, United States

**Keywords:** graph convolutional network, Human Connectome Project, KL divergence, persistent homology, structural and functional brain connectivity, topological data analysis, Wasserstein distance

## Abstract

**Introduction:**

Understanding the relationship between structural and functional brain connectivity remains a fundamental challenge in network neuroscience. While structural connectivity constrains neural interactions, functional connectivity emerges from complex, nonlinear dynamics that are not fully explained by anatomical wiring alone. Existing graph-based and deep learning approaches often focus on pairwise relationships, thereby overlooking higher-order topological organization in brain networks.

**Methods:**

We propose TopoGCN, a topology-informed graph convolutional network that integrates topological data analysis (TDA) into structure—function prediction. Persistent homology is used to extract multiscale topological features from structural connectomes, which are incorporated as node-level inputs. Additionally, a topology-aware loss function is incorporated to enforce preservation of higher-order structures in predicted functional networks. The model is evaluated on Human Connectome Project data (*n* = 998) using both edge-wise metrics and topological measures, including motif distributions, Betti curves, and Wasserstein distances between persistence diagrams.

**Results:**

TopoGCN achieves competitive accuracy in reconstructing functional connectivity while significantly improving the preservation of higher-order network organization. Compared to state-of-the-art models, it yields lower KL divergence for node strength and motif distributions, and smaller Wasserstein distances between persistence diagrams, indicating better recovery of multiscale topological structures. Furthermore, hypothesis tests on Betti curves show no significant differences between observed and predicted networks, demonstrating strong agreement in both connected-component and cycle-based topology.

**Discussion:**

Our findings show that incorporating persistent-homology information into graph learning improves the modeling of structure-function relationships in the human brain. By preserving multiscale topological features beyond pairwise connections, TopoGCN provides an improved framework for topology-aware connectome prediction and offers new opportunities for studying brain organization and individual variability.

## 1 Introduction

Understanding the relationship between structural connectivity and functional connectivity remains a central challenge in network neuroscience. Structural connectivity describes the anatomical connections between brain regions through white-matter pathways, typically estimated using diffusion magnetic resonance imaging (dMRI) and tractography. In contrast, functional connectivity characterizes statistical dependencies between neural signals, commonly measured using functional magnetic resonance imaging (fMRI) (Sporns et al., 2005). Although structural connections provide the physical substrate that constrains neural communication, functional interactions emerge from complex dynamical processes that cannot be fully explained by anatomical wiring alone. Consequently, predicting functional connectivity from structural connectivity has become an important problem in computational neuroscience, with applications ranging from understanding brain organization to identifying biomarkers of neurological and psychiatric disorders. Early studies demonstrated that structural connectivity explains a substantial proportion of the variance in resting-state functional connectivity, establishing a fundamental link between brain anatomy and function (Honey et al., 2009; Meier et al., 2016; Honey et al., 2010; Suárez et al., 2020). However, the relationship remains incomplete and highly nonlinear, motivating the development of increasingly sophisticated computational models capable of capturing the mechanisms underlying structure–function coupling.

Recent advances in machine learning have significantly improved the modeling of structure–function relationships. Traditional approaches relied on statistical regression models, diffusion processes, or dynamical systems that simulated neural activity on structural networks (Zamani Esfahlani et al., 2022; Honey et al., 2009). More recently, graph neural networks (GNNs) have emerged as a powerful framework for connectome analysis because they naturally accommodate graph-structured data and can learn nonlinear interactions among brain regions. By treating the structural connectome as a graph, GNNs enable information to propagate through anatomical pathways while simultaneously learning latent representations that are predictive of functional organization. Several recent studies have demonstrated the effectiveness of GNN-based models for functional connectome prediction. For example, (Neudorf et al. 2022) developed a graph-network architecture that predicts functional connectivity and network centrality measures directly from structural connectivity, showing substantial improvements over conventional approaches. (Li et al. 2022) proposed a graph convolutional encoder-decoder framework that learns multiscale representations of structural networks to reconstruct functional connectivity. More recently, (Chen et al. 2024) demonstrated that graph neural networks can capture both group-level and individual-specific components of structure–function coupling, highlighting their potential for subject-level connectome prediction. Similarly, several other neural network-based approaches have been developed to learn non-linear mappings from structural to functional connectivity, including feedforward neural networks (Lin et al., 2021), graph-based generative adversarial networks (Etemadyrad et al., 2022), and attention-based multiscale graph neural networks (Oota et al., 2024). Despite these advances, existing approaches are largely driven by edge-wise reconstruction objectives and primarily focus on pairwise connectivity relationships.

A major limitation of current structure–function prediction methods is that they often neglect higher-order network organization. Brain networks exhibit complex topological structures that extend beyond individual connections and local neighborhoods. Examples include cycles, cavities, motifs, and mesoscale patterns that emerge from coordinated interactions among multiple brain regions. Such higher-order structures have been shown to play important roles in neural communication, cognitive processing, and network resilience (Saggar et al., 2018; Das et al., 2023; Songdechakraiwut and Chung, 2023). Conventional graph-theoretic measures can characterize certain aspects of network organization, but they often depend on arbitrary thresholding choices and may fail to capture multiscale topological phenomena. As a result, two networks with similar edge-level properties can exhibit substantially different higher-order organization. Topological data analysis (TDA) provides a mathematically rigorous framework for characterizing higher-order organization in complex networks. Persistent homology, one of the principal tools in TDA, quantifies the appearance and disappearance of topological features across multiple scales and evaluates these changes through topological summaries, such as persistence diagrams, persistence barcodes, and Betti curves (Zomorodian and Carlsson, 2005; Carlsson, 2009; Edelsbrunner and Harer, 2010; Boissonnat et al., 2018). The integration of topological information into graph learning has recently emerged as a promising direction in geometric deep learning. Existing studies have shown that topological summaries can improve graph classification, network comparison, and representation learning by providing information that is complementary to conventional graph features (Songdechakraiwut and Chung, 2023; Carrière et al., 2020; Moor et al., 2020; Chung et al., 2019b).

In this study, we propose TopoGCN, a topology-informed graph convolutional network for predicting functional connectivity from structural connectivity. The proposed framework incorporates topological information in two complementary ways. First, persistent homology derived from graph filtrations of structural connectomes is used to construct node-level topological features that quantify each brain region's participation in persistent cycle structures. These features are incorporated directly into the graph convolutional network encoder to enrich regional representations with multiscale topological information. Second, we introduce a topology-aware loss function that encourages the predicted functional connectome to preserve the multiscale topological organization of the observed functional network. By combining topology-informed node representations with topology-aware optimization, TopoGCN simultaneously models local structural interactions and higher-order network organization.

We evaluate the proposed framework using paired structural and functional connectomes from the Human Connectome Project and compare its performance with several state-of-the-art graph-based structure–function prediction models. Beyond conventional edge-level measures, we assess the ability of competing methods to preserve higher-order network organization using motif distributions, persistence diagrams, Wasserstein distances, and Betti curves. Our results demonstrate that TopoGCN achieves improved preservation of multiscale topological structure while maintaining competitive edge-wise prediction accuracy. These findings suggest that incorporating persistent-homology information into graph convolutional networks provides a principled approach for modeling the complex relationship between structural and functional brain networks and offers a new direction for topology-aware connectome prediction.

## 2 Dataset

This study uses paired structural and functional connectivity data from the Human Connectome Project, following the dataset used in (Neudorf et al. 2022). The dataset consists of diffusion MRI and resting-state fMRI data from 998 healthy adult participants. For each subject, the brain is represented as a connectivity network comprising *p* = 116 anatomically defined brain regions, which constitute the nodes of the network. Accordingly, the structural and functional connectivity matrices are both of dimension 116 × 116 and are defined over the same set of brain regions. Structural connectivity is estimated from diffusion MRI tractography and summarized using streamline-count-based connection strengths. Functional connectivity is computed from resting-state fMRI time series using pairwise Pearson correlations between regional signals. Thus, each subject is represented by a structural connectivity matrix and a corresponding functional connectivity matrix defined over the same set of brain regions. Figure 1 presents representative structural and functional connectivity matrices together with their thresholded corresponding network visualizations for one subject.

**Figure 1:**
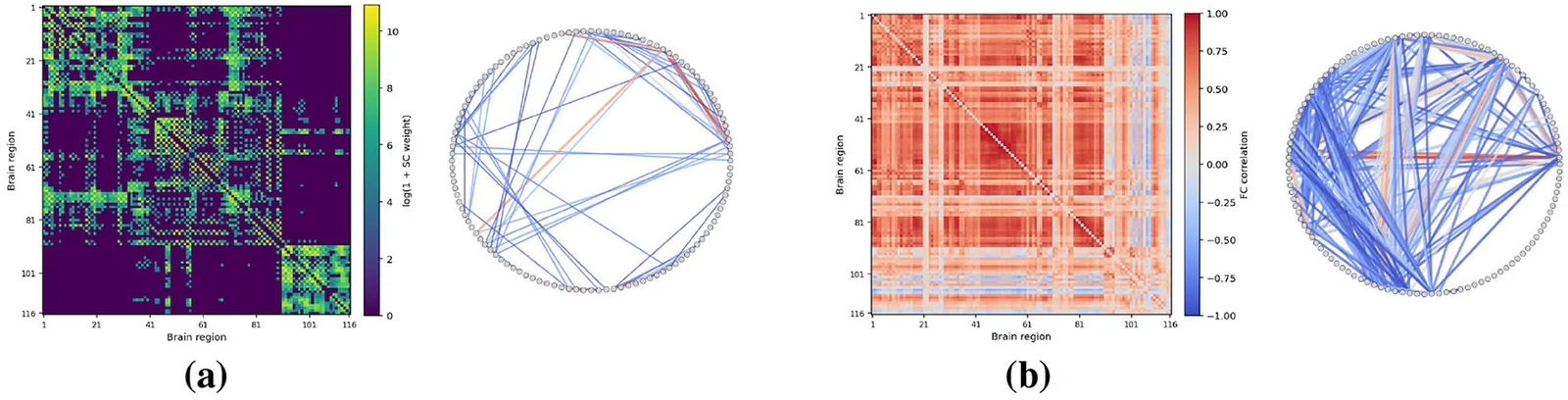
Representative structural and functional connectomes for one individual. **(a)** Structural connectivity. **(b)** Functional connectivity.

For each subject *i*, we denote the structural connectivity matrix by $S_{i}\in {\mathbb{R}}^{p\times p}$ and the functional connectivity matrix by $F_{i}\in {\mathbb{R}}^{p\times p}$, where *p* denotes the number of brain regions. Our goal is to learn a mapping from structural connectivity to functional connectivity,


$$\begin{array}{l} {\hat{F}}_{i}=f_{\theta }\left(S_{i},X_{i}\right), \end{array}$$ (1)

where *f*_θ_ denotes our graph convolutional network with parameters θ, *X*_*i*_ denotes the node feature matrix for subject *i*, and ${\hat{F}}_{i}$ denotes the predicted functional connectivity matrix.

## 3 Methods

### 3.1 TDA for brain connectivity networks

#### 3.1.1 Persistent homology

Topological data analysis (TDA) provides a multiscale framework for characterizing the organization of complex data beyond pairwise or local graph summaries. This perspective is especially relevant for brain connectivity analysis, where structural and functional connectomes are commonly represented as weighted networks. However, many standard graph summaries depend on the choice of a single threshold. While graph-based methods describe relationships between pairs of regions, algebraic-topological approaches extend this representation by encoding higher-order interactions through *simplicial complexes* (Carlsson, 2009; Carlsson and Gabrielsson, 2020). The basic building block of a simplicial complex is a simplex: a *k*-simplex is determined by *k*+1 vertices. Thus, a single node forms a 0-simplex, an edge forms a 1-simplex, three mutually connected regions form a 2-simplex represented by a filled triangle, and larger mutually connected groups give rise to higher-dimensional simplices [similar definitions of *clique-complex* (Giusti et al., 2015) are defined in Section 3.1.2]. A filtration is then formed by arranging these simplicial complexes in a nested sequence as a filtration parameter varies, so that simplices included at one level remain present at subsequent levels. *Persistent homology* then tracks the emergence and disappearance of connected components, cycles, and higher-order cavities across across this filtration, providing a summary of network organization across multiple connectivity scales (Chazal and Michel, 2021; Otter et al., 2017; Dey and Gel, 2026).

In the study of brain networks, persistent homology provides a powerful framework for quantifying global and mesoscale connectivity patterns that are not fully captured by conventional graph-theoretic measures. This framework is particularly well suited to connectomic data, which exhibit higher-order topological structures, such as cycles and cavities, reflecting coordinated interactions among multiple brain regions beyond pairwise connectivity. By tracking the emergence and persistence of these topological features across a *filtration process*, persistent homology provides a multiscale characterization of network topology. This capability is especially advantageous for connectome analysis, where conventional graph summaries can be sensitive to arbitrary choices of network thresholding (Sizemore et al., 2019).

Several filtration strategies have been developed in the TDA literature to construct simplicial complexes and compute persistent homology from complex networks, including Vietoris–Rips (Edelsbrunner and Harer, 2010), Čech (Ghrist, 2008), and clique-complex filtration (Giusti et al., 2015, 2016; Aktas et al., 2019) filtrations. In this study, we adopt the *clique-complex filtration*, which is particularly well suited for weighted brain network data because it constructs the filtration directly from the network connectivity and its associated edge weights.

#### 3.1.2 Graph filtration and clique complex

To characterize the multiscale organization of the structural connectome, we represent each subject's structural connectivity matrix as a weighted brain network and examine how its connectivity pattern evolves as progressively weaker structural connections are introduced. For subject *i*, let $S_{i}\in {\mathbb{R}}^{p\times p}$ denote the structural connectivity matrix, where *p* = 116 denotes the number of brain regions. We represent *S*_*i*_ as a weighted undirected graph *G*_*i*_ = (*V, E*_*i*_, *w*_*i*_) where *V* = {*v*_1_, …, *v*_*p*_} is the set of brain regions. For any two brain regions *u, v* ∈ *V*, the matrix entry *S*_*i*_(*uv*) represents the structural connectivity strength between regions *u* and *v*. Accordingly, we define the edge set as *E*_*i*_ = {(*u, v*):*S*_*iuv*_ > 0, *u* < *v*} and assign each edge the weight *w*_*i*_(*u, v*) = *S*_*iuv*_.

We next transform the structural connection weights into filtration values, which determine the order in which edges are introduced into the network. Specifically, for each (*u, v*) ∈ *E*_*i*_ we define,


$$\begin{array}{l} \tau_{i}\left(u,v\right)=1-\frac{w_{i}\left(u,v\right)}{\max_{\left(a,b\right)\in E_{i}}w_{i}\left(a,b\right)}. \end{array}$$

Under this transformation, stronger structural connections receive smaller filtration values and therefore enter the filtration earlier. For a given filtration parameter α ∈ [0, 1], we retain all edges whose filtration values satisfy τ_*i*_(*u, v*) ≤ α. This gives the edge set *E*_*i*_(α) = {(*u, v*) ∈ *E*_*i*_:τ_*i*_(*u, v*) ≤ α} and the resulting thresholded graph is defined as *G*_*i*_(α) = (*V, E*_*i*_(α)). Thus, when α is small, the graph contains only the strongest structural connections. As α increases, progressively weaker connections are added while all previously included edges are retained. Consequently, for α_1_ < α_2_ < … < α_*n*_, we obtain the nested sequence


$$\begin{array}{l} G_{i}\left(\alpha_{1}\right)\subseteq G_{i}\left(\alpha_{2}\right)\subseteq \ldots \subseteq G_{i}\left(\alpha_{n}\right), \end{array}$$

which defines the graph filtration of the structural connectome (Petri et al., 2013; Aktas et al., 2019; Chung et al., 2024).

Figure 2 provides an illustration of the graph filtration defined above for a representative subset of brain regions from the Automated Anatomical Labeling (AAL) atlas, a commonly used brain parcellation that divides the brain into anatomically defined regions. In panel (a), the filtration level is set to α = 0.10, so that the graph *G*_*i*_(0.10) = (*V, E*_*i*_(0.10)) contains only edges satisfying τ_*i*_(*u, v*) ≤ 0.10. These edges correspond to the strongest structural connections and therefore form a relatively sparse network. Increasing the filtration level to α = 0.25 in panel (b) expands the edge set to *E*_*i*_(0.25) = {(*u, v*) ∈ *E*_*i*_:τ_*i*_(*u, v*) ≤ 0.25}, where *E*_*i*_(0.10)⊆*E*_*i*_(0.25) and additionally includes connections satistying 0.10 < τ_*i*_(*u, v*) ≤ 0.25. The same progression is observed in panels (c) and (d), corresponding to α = 0.40 and α = 0.60, respectively, as additional weaker connections enter the graph. Thus, the four panels represent the nested sequence *G*_*i*_(0.10)⊆*G*_*i*_(0.25)⊆*G*_*i*_(0.40)⊆*G*_*i*_(0.60), illustrating how the structural network becomes progressively denser as the filtration parameter increases.

**Figure 2:**
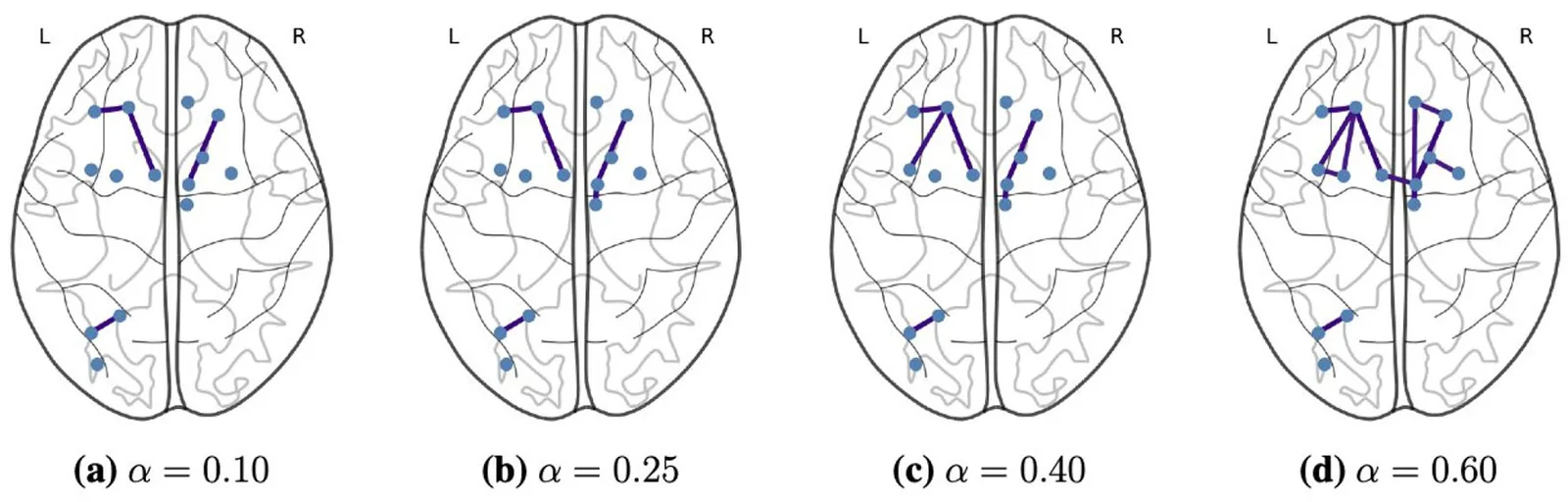
Illustration of the structural graph filtration for a representative subset of AAL brain regions. Panels **(a–d)** show the thresholded graphs *G*_*i*_(0.10), *G*_*i*_(0.25), *G*_*i*_(0.40), and *G*_*i*_(0.60), respectively.

The thresholded graph *G*_*i*_(α) describes the pairwise connectivity structure of the brain at each filtration level α. To characterize higher-order patterns formed by groups of mutually connected brain regions, we next associate each thresholded graph with its clique complex, *K*_*i*_(α) = Cl*G*_*i*_(α). A clique is a subset of vertices in which every pair of distinct vertices is connected by an edge in *G*_*i*_(α). Accordingly, the clique complex is defined as


$$\begin{array}{l} K_{i}\left(\alpha \right)=\left\{\sigma \subseteq V:\left(u,v\right)\in E_{i}\left(\alpha \right)\text{ for every distinct}u,v\in \sigma \right\}. \end{array}$$ (2)

Here, σ⊆*V* denotes any subset of brain regions. The subset σ is included in *K*_*i*_(α) only when every pair of regions in σ is connected in the thresholded graph *G*_*i*_(α). Thus, *K*_*i*_(α) is the collection of all fully connected subsets of brain regions present at filtration level α. For example, if three brain regions are mutually connected in *G*_*i*_(α), they form a three-vertex clique and are represented by the corresponding 2-simplex in *K*_*i*_(α). If any one of the three pairwise connections is absent, the corresponding 2-simplex is not included (Giusti et al., 2015).

In this way, the clique complex retains the vertices and edges of *G*_*i*_(α) while also representing fully connected groups of three or more brain regions through higher-dimensional simplices. The construction therefore provides a simplicial representation of the connectivity pattern observed at each filtration level.

As the filtration parameter increases, the thresholded graphs become nested and, consequently, their associated clique complexes also satisfy


$$\begin{array}{l} K_{i}\left(\alpha_{1}\right)\subseteq K_{i}\left(\alpha_{2}\right)\subseteq \cdots \subseteq K_{i}\left(\alpha_{n}\right), \end{array}$$ (3)

on which persistent homology is computed (Giusti et al., 2016; Sizemore et al., 2018). In this study, we focus on 0- and 1-dimensional persistent homology, *H*_0_ and *H*_1_, which characterize the evolution of connected components and one-dimensional holes (cycles), respectively, across filtration scales.

The graph filtration and its associated nested clique complexes provide the basis for the complementary topological summaries used in this study. First, the persistent *H*_1_ cycles identified from the clique-complex filtration of the structural connectome are used to derive the node-level topological features described in Section 3.1.3, which are subsequently incorporated into the TopoGCN model. We further use persistence diagrams and Betti curves as complementary summaries to assess the topological agreement between the observed and TopoGCN-predicted functional connectivity networks.

The evolution of the topological features across the filtration scale, α_1_ < α_2_ < … < α_*n*_, is summarized in a *persistence diagram*. For subject *i*, the persistence diagram


$$\begin{array}{l} D_{h}^{\left(i\right)}=\left\{\left(b_{ij},d_{ij}\right)\in {\mathbb{R}}^{2}:b_{ij}<d_{ij},\;j=1,\ldots ,m_{i}\right\}, \end{array}$$ (4)

represents the multi-set of birth and death times of the homology feature *H*_*h*_, where *h* is either 0 or 1, and *m*_*i*_ is the total number of *h*-dimensional topological features. Here, *b*_*ij*_ denotes the filtration level at which the *j*th feature appears, and *d*_*ij*_ denotes the filtration level at which it disappears. The persistence, or lifetime, of the *j*th feature is defined as *L*_*ij*_ = *d*_*ij*_−*b*_*ij*_. Features with larger lifetimes persist across a wider range of filtration values and therefore represent more prominent homology features in the structural connectome.

We also compute Betti curves to describe how the topological features of the structural connectome evolve across filtration levels. For a given filtration level α, the Betti-0, $\beta_{0}^{\left(i\right)}\left(\alpha \right)$, is defined as the number of connected components, while Betti-1, $\beta_{1}^{\left(i\right)}\left(\alpha \right)$, is defined as the number of independent cycles. Evaluating Betti-0 and Betti-1 across filtration levels α_1_ < α_2_ < … < α_*n*_ yields the β_0_ and β_1_ Betti curves, i.e., ${\text{β}}_{h}^{i}=\left(\beta_{h}^{i}\left(\alpha_{1}\right),\beta_{h}^{i}\left(\alpha_{2}\right),\ldots ,\beta_{h}^{i}\left(\alpha_{n}\right)\right)$, *h* = 0, 1, which describe how number of connected-components and cycles evolve across the filtration (Edelsbrunner and Harer, 2010; Boissonnat et al., 2018; Chung et al., 2019a).

Similar to the structural connectivity matrix *S*_*i*_, graph filtration can also be applied to the functional connectivity matrix $F_{i}\in {\mathbb{R}}^{p\times p}$ to characterize its topological organization across multiple scales. This filtration enables the computation of higher-order topological summaries, such as persistence diagrams and Betti curves, which capture the emergence and evolution of topological features throughout the filtration process.

#### 3.1.3 Node-level topological features

In this section, we focus on one-dimensional persistent homology and derive node-level topological features from the persistent *H*_1_ cycles in the structural connectome. These persistent cycles are identified from the clique-complex filtration {_*K*_*i*_(α)}α ∈ [0, 1]_ constructed in the Section 3.1.2. As the filtration parameter increases, edges and the corresponding higher-dimensional simplices are progressively added to the complex, and persistent homology tracks the birth and death of *H*_1_ cycle across the filtration. Specifically, we characterize how individual brain regions participate in these persistent cycle structures. Since persistent homology describes cycle-like organization at the network level, whereas the graph convolutional network requires features defined for individual brain regions, we summarize the persistent *H*_1_ cycle information at the node level using two measures: node persistence and node frequency.

Let *C*_*ij*_⊆*V* denote the set of brain regions contained in the *j*th persistent *H*_1_ cycle for subject *i*, and let *L*_*ij*_ = *d*_*ij*_−*b*_*ij*_ denote its lifetime. The node persistence of region *v* ∈ *V* is defined as


$$\begin{array}{l} {\text{NP}}_{iv}=\frac{1}{D_{i}}\overset{m_{i}}{\underset{j=1}{\sum }}L_{ij}I\left\{v\in C_{ij}\right\}, \end{array}$$

where *D*_*i*_ is a subject-specific normalizing constant defined by $D_{i}=\underset{j}{\max}d_{ij}-\underset{j}{\min}b_{ij}$, and *I*{·} denotes the indicator function, with *I*{*v* ∈ *C*_*ij*_} = 1 if region *v* belongs to the *j*th persistent *H*_1_ cycle and 0 otherwise. Thus, node persistence measures the total persistence of the cycle-like structures containing region *v*. A larger value indicates that the region participates in structures that remain present over a wider range of filtration levels.

In addition to node persistence, we define node frequency as the number of persistent *H*_1_ cycles in which a region *v* participates:


$$\begin{array}{l} {\text{NF}}_{iv}=\overset{m_{i}}{\underset{j=1}{\sum }}I\left\{v\in C_{ij}\right\}. \end{array}$$

Thus, whereas node persistence weights participation by cycle lifetime, node frequency records how often region *v* appears in persistent cycle structure. In this way, node persistence reflects the strength of a region's involvement in persistent topological organization, whereas node frequency reflects how frequently the region participates in such structures. The two measures therefore provide complementary region-level summaries of persistent cycle organization. We use the structural connectivity matrix $S_{i}\in {\mathbb{R}}^{p\times p}$ to construct the clique-complex filtration and apply persistent homology to identify the persistent *H*_1_ cycles across the filtration. The participation of each brain region in these cycles is then summarized through node persistence and node frequency, resulting in a *p*×2 node level representation across the *p* brain regions.

Once node persistence and node frequency are computed for all brain regions, they are combined with node identity features and used as inputs to the graph convolutional network. For subject *i*, let ${\text{NP}}_{i}\in {\mathbb{R}}^{p\times 1}$ and ${\text{NF}}_{i}\in {\mathbb{R}}^{p\times 1}$ denote the column vectors of node persistence and node frequency values across the *p* brain regions, respectively. The node feature matrix is then defined as


$$\begin{array}{l} X_{i}=\left[I_{p}|{\text{NP}}_{i}|{\text{NF}}_{i}\right], \end{array}$$ (5)

where $I_{p}\in {\mathbb{R}}^{p\times p}$ denotes the identity matrix and [·∣·] denotes column-wise feature concatenation. Consequently, $X_{i}\in {\mathbb{R}}^{p\times \left(p+2\right)}$. For *p* = 116, each brain region is therefore represented by 118 input features, consisting of 116 node-identity features together with one node-persistence and one node-frequency feature.

### 3.2 Topology-informed graph convolutional network (TopoGCN)

#### 3.2.1 TopoGCN architecture

Using the weighted structural graph together with these topology-informed node features *X*_*i*_, we now define the proposed graph convolutional network architecture for predicting subject-specific functional connectivity. For subject *i*, let $A_{i}\in {\mathbb{R}}^{p\times p}$ denote the weighted adjacency matrix derived from the structural connectivity matrix *S*_*i*_. The entries of *A*_*i*_ represent the structural connection strengths between brain regions and define the graph over which message passing is performed. Together, *A*_*i*_ and the node feature matrix *X*_*i*_ (Equation 5) form the input to our proposed graph convolutional network.

We initialize the node representations for subject *i* as $H_{i}^{\left(0\right)}=X_{i}$, where $H_{i}^{\left(0\right)}\in {\mathbb{R}}^{p\times \left(p+2\right)}$ and each row corresponds to the initial feature representation of one brain region. To allow information to propagate through the structural connectome, we apply graph convolutional layers. Following the graph convolutional formulation commonly used in graph neural networks (Kipf and Welling, 2016), we first augment the adjacency matrix with self-loops, ${\hat{A}}_{i}=A_{i}+I_{p},$ where *I*_*p*_ is the *p*×*p* identity matrix. Adding self-loops allows each brain region to retain its own information while also receiving information from structurally connected neighboring regions.

The degree matrix of the augmented structural graph is defined as ${\hat{D}}_{i}\left(u,u\right)=\overset{p}{\underset{s=1}{\sum }}{\hat{A}}_{i}\left(u,v\right)$ so that each diagonal entry ${\hat{D}}_{i}\left(u,u\right)$ gives the total connection strength of region *u* after self-loops are included. We then define the normalized structural propagation matrix as ${\tilde{A}}_{i}={\hat{D}}_{i}^{-1/2}{\hat{A}}_{i}{\hat{D}}_{i}^{-1/2}.$ This normalization stabilizes message passing more consistently across brain regions and reduces the influence of highly connected regions during aggregation. Using the normalized propagation matrix, the node representations are updated through a sequence of graph convolutional layers. At layer ℓ, the node representations are updated according to


$$\begin{array}{l} H_{i}^{\left(\ell +1\right)}=\phi \left({\tilde{A}}_{i}H_{i}^{\left(\ell \right)}W^{\left(\ell \right)}\right),\;\ell =0,\ldots ,L-1. \end{array}$$

where *W*^(ℓ)^ is the learnable weight matrix at layer ℓ, and ϕ(·) denotes a nonlinear activation function. In our implementation, we use a parametric rectified linear unit after each graph-convolutional layer. After *L* graph-convolutional layers, we obtain the final node embedding matrix $Z_{i}=H_{i}^{\left(L\right)},$ where each row of *Z*_*i*_ represents the learned embedding of one brain region. Because these embeddings are learned through message passing on the subject-specific structural connectome, they capture both the region's own topology-informed features and information aggregated from its neighboring brain regions.

The final node embeddings are then used to predict pairwise functional connectivity between brain regions. Let *z*_*iu*_ and *z*_*iv*_ denote the learned embeddings of brain regions *u* and *v*, corresponding to the *u*th and *v*th rows of *Z*_*i*_, respectively. For each pair of regions, we form a pairwise representation by concatenating their node embeddings, *e*_*iuv*_ =[*z*_*iu*_|*z*_*iv*_], where [·|·] denotes vector concatenation. This pairwise representation is then passed to a multilayer perceptron (MLP) decoder to estimate the functional connectivity between the two regions, ${\hat{F}}_{i}\left(u,v\right)=d_{\theta }\left(e_{iuv}\right),\;1\leq u<v\leq p,$ where *d*_θ_(·) denotes the decoder with learnable parameters θ. In this way, the decoder learns a non-linear mapping from pairs of node embeddings to functional connectivity strengths.

Because functional connectivity matrices are symmetric, we predict only the upper-triangular entries of the matrix and exclude the diagonal. For each pair of regions *u*<*v*, the decoder estimates a single functional connectivity value ${\hat{F}}_{i}\left(u,v\right)$, where 1 ≤ *u*<*v* ≤ *p*. The predicted values are then placed into the upper triangle of the functional connectivity matrix, and the lower triangle is obtained by symmetry, ${\hat{F}}_{i}\left(v,u\right)={\hat{F}}_{i}\left(u,v\right)$. The diagonal entries are set to zero, ${\hat{F}}_{i}\left(u,u\right)=0$. This produces the full predicted functional connectivity matrix ${\hat{F}}_{i}\in {\mathbb{R}}^{p\times p}$ for subject *i*. Figure 3 illustrates the architecture of the proposed TopoGCN for predicting functional connectivity from structural connectivity.

**Figure 3:**
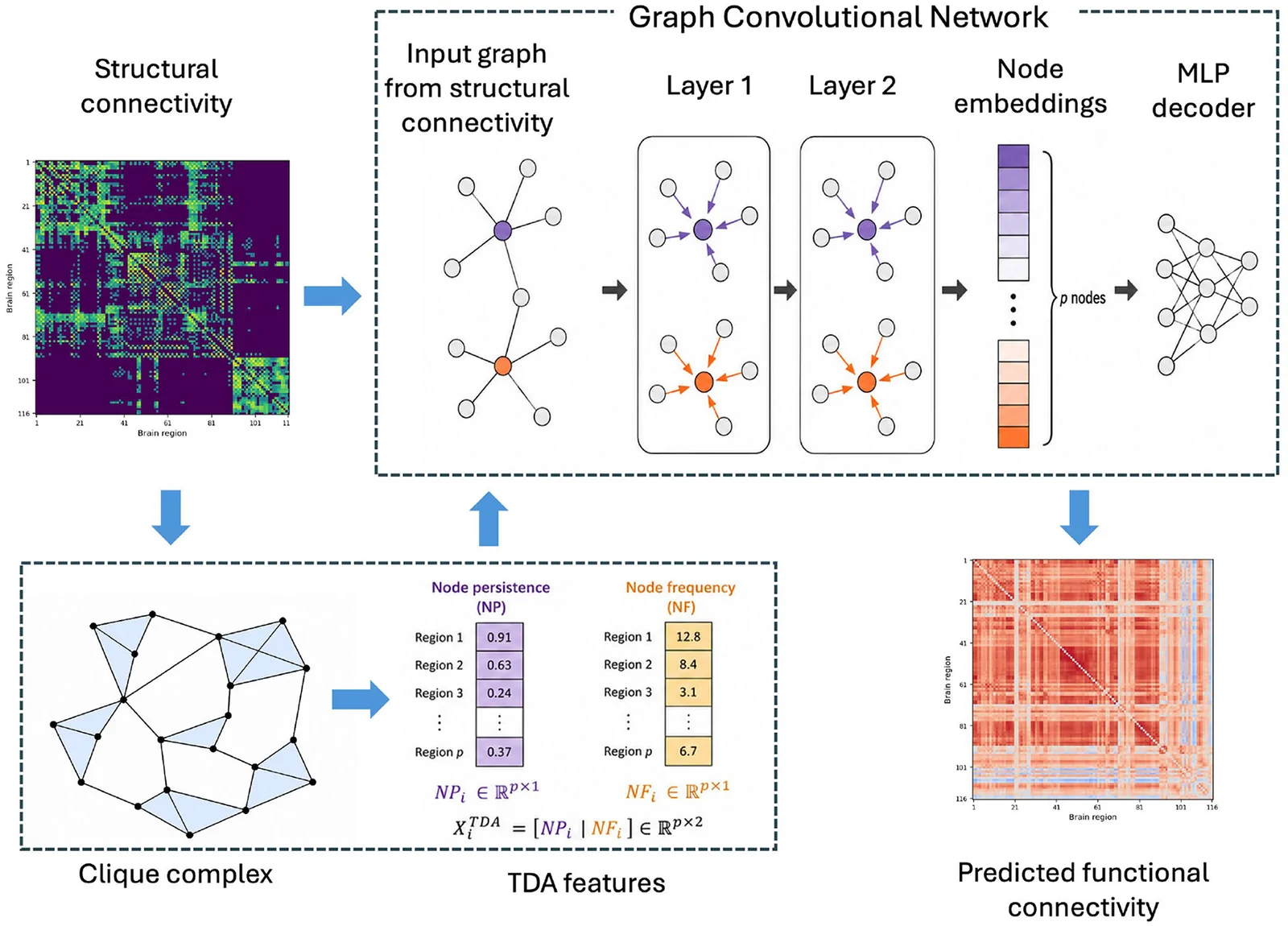
Architecture of the proposed TopoGCN.

#### 3.2.2 TopoGCN loss function

We train the proposed graph convolutional network in a supervised manner by minimizing a loss function over the training subjects. In graph neural networks, the loss function measures the discrepancy between the predicted output and the observed target. This discrepancy provides the signal used to optimize the model parameters through backpropagation (Scarselli et al., 2008; Kipf and Welling, 2016). In a standard supervised graph neural network, the learning objective can be written as


$$\begin{array}{l} {\text{Θ}}^{*}=\arg\underset{\text{Θ}}{\min}\frac{1}{\left|T\right|}\underset{i\in T}{\sum }L\left(y_{i},f_{\text{Θ}}\left(G_{i},X_{i}\right)\right), \end{array}$$ (6)

where Θ denotes the collection of trainable parameters, T denotes the training subjects, *f*_Θ_(*G*_*i*_, *X*_*i*_) is the model prediction for subject *i*, *y*_*i*_ is the corresponding observed target, and L(·,·) is a task-specific loss function.

In our setting, the training objective for each subject *i* combines two complementary components. The first component is the edge-reconstruction loss, which encourages the predicted functional connectivity matrix to match the observed functional connectivity matrix at the level of individual pairwise connections. The second component is the topology-aware regularization loss, to preserve multiscale topological organization of the observed functional network. Accordingly, we define the subject-specific loss as


$$\begin{array}{l} L^{\left(i\right)}=L_{\text{edge}}^{\left(i\right)}+{\text{λ}}_{\text{topo}}L_{\text{topo}}^{\left(i\right)}, \end{array}$$ (7)

where $L_{\text{edge}}^{\left(i\right)}$ denotes the edge-wise reconstruction loss, $L_{\text{topo}}^{\left(i\right)}$ denotes the topology-aware loss, and λ_topo_ controls the contribution of the topological regularization term. The model parameters are then estimated by minimizing the average subject-specific loss over the training set following the general supervised learning objective in Equation 6. The $L_{\text{edge}}^{\left(i\right)}$ measures the difference between the predicted and observed functional connectivity matrices at the level of individual pairwise connections. In our implementation, this loss is defined as the mean squared error over all unique off-diagonal entries:


$$\begin{array}{l} L_{\text{edge}}^{\left(i\right)}=\frac{1}{q}\underset{1\leq u<v\leq p}{\sum }{\left({\hat{F}}_{i}\left(u,v\right)-F_{i}\left(u,v\right)\right)}^{2}, \end{array}$$ (8)

where *q* = *p*(*p*−1)/2 is the number of unique off-diagonal connections in the functional connectivity matrix. This term encourages the model to reconstruct the observed functional connectome accurately at the edge level.

Although the $L_{\text{edge}}^{\left(i\right)}$ encourages close correspondence between predicted and observed functional connections at the pairwise level, it does not explicitly constrain the global topological organization of the predicted functional connectome. Following the approach of (Songdechakraiwut and Chung 2023), we incorporate a topological loss, $L_{\text{topo}}^{\left(i\right)}$, based on persistent homology to preserve the multiscale topological structure of the functional connectome. This loss compares the persistent topological summaries of the predicted and observed functional connectomes across the filtration.

For each subject *i*, we transform the observed and predicted functional connectivity matrices into distance-based representations so that stronger functional connections enter the filtration earlier. For each pair of brain regions *u*<*v*, we define $d_{i}\left(u,v\right)=\sqrt{2\left(1-F_{i}\left(u,v\right)\right)}$ and ${\hat{d}}_{i}\left(u,v\right)=\sqrt{2\left(1-{\hat{F}}_{i}\left(u,v\right)\right)}$. Under this transformation, stronger functional correlations correspond to smaller distances, so strongly associated brain-region pairs appear earlier in the filtration. We then apply Kruskal's algorithm (Kruskal, 1956) to the complete weighted graph defined by the pairwise distances, processing the edges in increasing order of their filtration values. This separates the edges into two groups. Edges that connect previously disconnected components are included in the minimum spanning tree (Cormen et al., 2022) and form the 0-dimensional birth set. In contrast, edges whose endpoints are already connected create cycles and form the 1-dimensional death set. For subject *i*, we denote the resulting birth and death sets of the observed functional connectome by


$$\begin{array}{l} B_{i}=\left\{b_{i1},b_{i2},\ldots ,b_{i,p-1}\right\},\;D_{i}=\left\{d_{i1},d_{i2},\ldots ,d_{i,q-p+1}\right\}, \end{array}$$

where *p* is the number of brain regions and *q* = *p*(*p*−1)/2 is the number of unique undirected edges. Similarly, from the predicted functional connectome we obtain the corresponding predicted birth and death sets


$$\begin{array}{l} {\hat{B}}_{i}=\left\{{\hat{b}}_{i1},{\hat{b}}_{i2},\ldots ,{\hat{b}}_{i,p-1}\right\},\;{\hat{D}}_{i}=\left\{{\hat{d}}_{i1},{\hat{d}}_{i2},\ldots ,{\hat{d}}_{i,q-p+1}\right\}. \end{array}$$

Using these ordered sequences, we define the normalized 0-dimensional topology loss, which measures the discrepancy between the observed and predicted birth set as $L_{0\text{D}}^{\left(i\right)}=\frac{1}{p-1}\overset{p-1}{\underset{k=1}{\sum }}{\left({\hat{b}}_{ik}-b_{ik}\right)}^{2}.$ Similarly, the 1-dimensional topology loss compares the observed and predicted death sequences and is defined as $L_{1\text{D}}^{\left(i\right)}=\frac{1}{q-p+1}\overset{q-p+1}{\underset{k=1}{\sum }}{\left({\hat{d}}_{ik}-d_{ik}\right)}^{2}.$ Finally, the subject-specific total topology-aware loss combines the 0-dimensional and 1-dimensional components as


$$\begin{array}{l} L_{\text{topo}}^{\left(i\right)}=L_{0\text{D}}^{\left(i\right)}+{\text{λ}}_{1\text{D}}L_{1\text{D}}^{\left(i\right)}, \end{array}$$ (9)

where λ_1D_ controls the relative contribution of the 1-dimensional term. In our implementation, we set λ_1D_ = 1, assigning equal weight to the 0 and 1-dimensional topology losses.

#### 3.2.3 Model training and implementation details

We train the proposed TopoGCN model using mini-batch gradient-based optimization (Robbins and Monro, 1951) on the training set. The dataset is divided into training, validation, and test subsets, with the validation set used for model selection. The test set is kept independent of model selection and is used only for the final evaluation, providing an unbiased assessment of model generalization. For each subject, the structural connectivity matrix is first symmetrized, and its diagonal entries are set to zero to exclude self-connections. Because the structural edge weights are based on streamline counts and exhibit a wide dynamic range, the off-diagonal entries are transformed using log(1+*x*). The model is trained for 400 epochs using the Adam optimizer (Kingma and Ba, 2014) with a learning rate of 0.001 and a mini-batch size of 2. The graph encoder consists of two graph convolutional layers, each with a hidden dimension of 256, while the edge decoder uses a hidden dimension of 64. At each iteration, the model parameters are updated by minimizing the loss function defined above. We experiment with different values of λ_topo_ and finally set the topology-loss weight to λ_topo_ = 0.1, as this value provides a stable balance between the edge-reconstruction and the topology-aware regularization term during training (Supplementary Section 1.1 provides a detailed description of the experiment, while Supplementary Table S1 reports the prediction performance for different values of λ_topo_). In addition, a small *L*_2_ regularization term (Hoerl and Kennard, 1970) is applied during optimization to reduce model complexity and limit overfitting. The model parameters corresponding to the lowest validation loss are retained for final evaluation on the test set, further reducing the risk of selecting a model that is overly fitted to the training data.

The complete training procedure for the proposed topology-induced graph convolutional network is summarized in Algorithm 1.

Algorithm 1 Training the topology-induced graph convolutional network. 
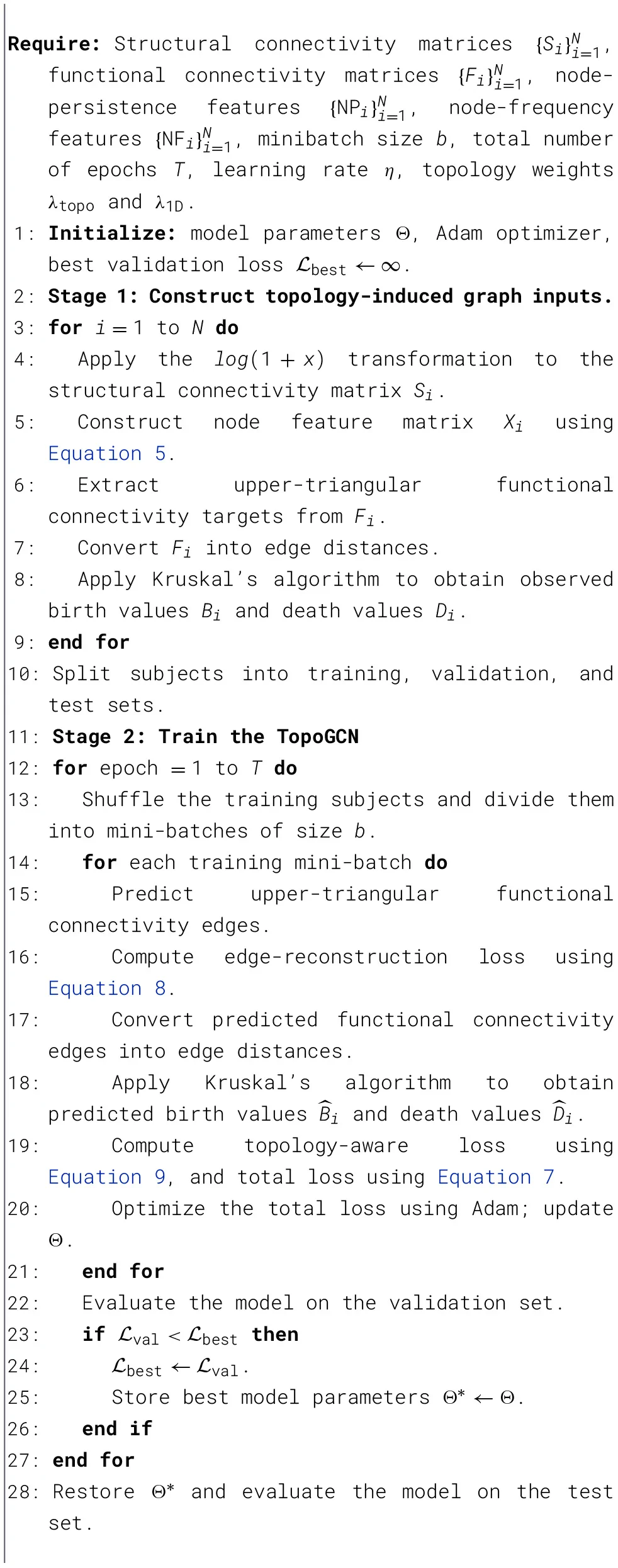

## 4 Performance measures

We evaluate the proposed TopoGCN model using network-level graph measures, and higher-order topological measures, e.g., node strength (weight) distribution, motif distribution, persistence diagram, and Betti curves. These evaluations assess the accuracy of individual functional connectivity predictions together with the preservation of the higher-order topological organization of the observed functional connectome. The full dataset consists of 998 subjects, which we divide into 698 training subjects, 99 validation subjects, and 201 test subjects. For a fair comparison, we evaluate all models on the same held-out test set of 201 subjects, and we adopt the same training and implementation protocol for the competing methods.

For each test subject *i* = 1, …, *N*, let ${\hat{F}}_{i}\in {\mathbb{R}}^{p\times p}$ denote the predicted functional connectivity matrix and let $F_{i}\in {\mathbb{R}}^{p\times p}$ denote the corresponding observed functional connectivity matrix. For a given performance metric *m*(·, ·), we define the pairwise subject-by-subject comparison matrix $c_{ij}^{\left(m\right)}=m\left({\hat{F}}_{i},F_{j}\right),i,j=1,\ldots ,N$, where $c_{ij}^{\left(m\right)}$ quantifies the agreement between the predicted matrix of subject *i* and the observed matrix of subject *j*. The diagonal entries $c_{ii}^{\left(m\right)}$ correspond to *matched* comparisons, whereas the off-diagonal entries $c_{ij}^{\left(m\right)}$, with *i*≠*j*, correspond to *mismatched* comparisons. Figure 4 illustrates this comparison framework.

**Figure 4:**
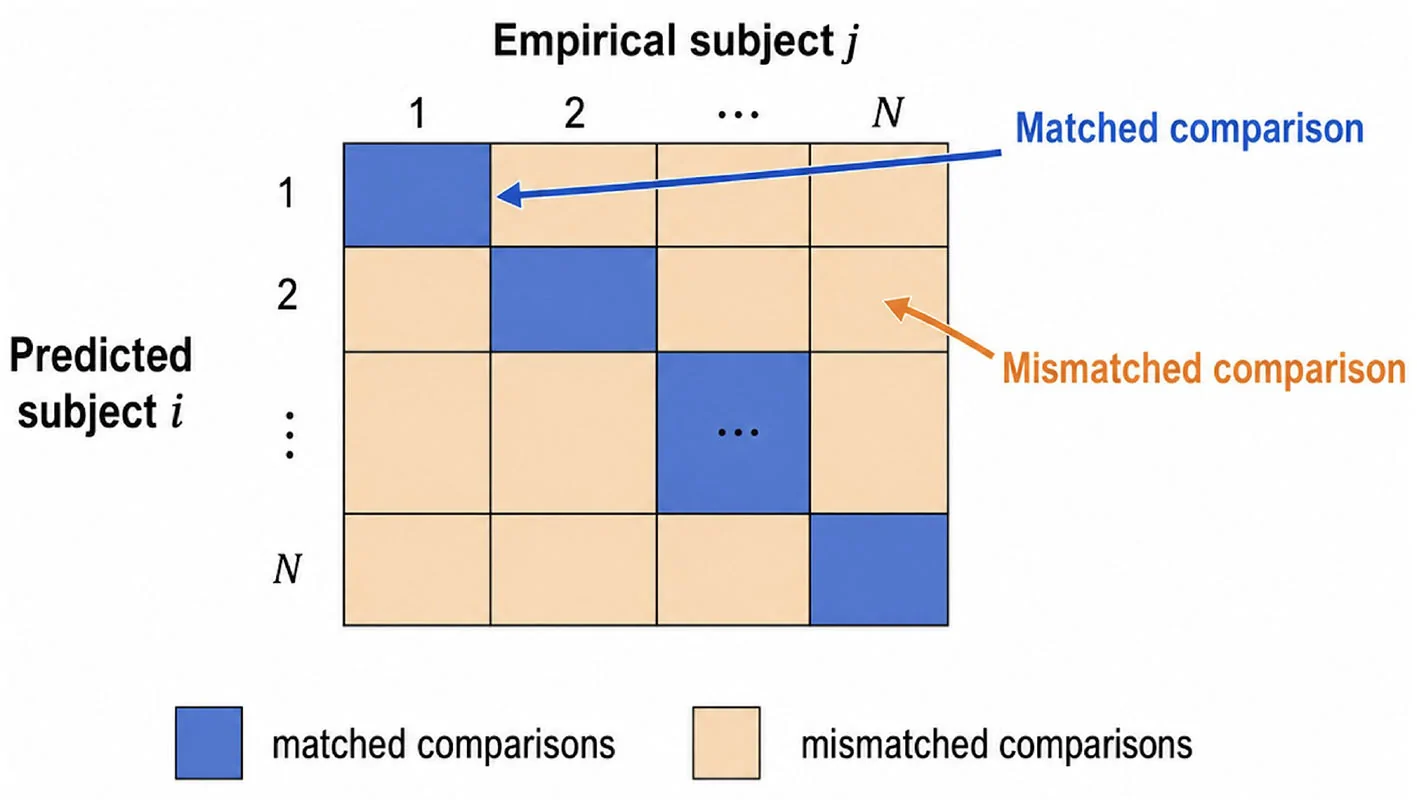
Comparison matrix.

Following (Chen et al. 2024), we summarized the group-common effect using the average mismatched comparison


$$\begin{array}{l} c_{\text{mismatch}}^{\left(m\right)}=\frac{1}{N\left(N-1\right)}\underset{i\neq j}{\sum }c_{ij}^{\left(m\right)} \end{array}$$

and the matched effect using the average diagonal comparison


$$\begin{array}{l} c_{\text{match}}^{\left(m\right)}=\frac{1}{N}\overset{N}{\underset{i=1}{\sum }}c_{ii}^{\left(m\right)} \end{array}$$

The quantity $c_{\text{mismatch}}^{\left(m\right)}$ represents the average agreement between the predicted functional connectivity matrix of one subject and the empirical matrices of other subjects, and therefore reflects group-common structure. In contrast, $c_{\text{match}}^{\left(m\right)}$ represents the average agreement between each predicted functional connectivity matrix and its corresponding empirical matrix, and therefore reflects matched individual-level agreement. The comparison between these two quantities indicates how well a model preserves subject-level organization beyond shared group structure.

Because the measures considered here are distance-based, smaller values indicate better agreement. We therefore define the individual-specific effect as


$$\begin{array}{l} c_{\text{individual}}^{\left(m\right)}=c_{\text{mismatch}}^{\left(m\right)}-c_{\text{match}}^{\left(m\right)}. \end{array}$$

Thus, a larger positive value of $c_{\text{individual}}^{\left(m\right)}$ indicates that the predicted functional connectivity matrix of a subject is, on average, closer to its own observed functional connectivity matrix than to the observed matrices of other subjects. In this sense, the individual-specific effect quantifies how well a model preserves subject-specific organization, that is, connectivity patterns that distinguish one subject from another rather than features that are common across the cohort.

### 4.1 Kullback–Leibler divergence

We apply the Kullback–Leibler (KL) divergence to compare distributional properties of predicted and observed functional connectivity networks. KL divergence quantifies the information loss incurred when one probability distribution is used to approximate another. For two discrete distributions *P*(*p*_ℓ_) and *Q*(*q*_ℓ_) defined on the same support, the KL divergence is


$$\begin{array}{l} D_{\text{KL}}\left(P||Q\right)=\underset{\ell }{\sum }p_{\ell }\log\frac{p_{\ell }}{q_{\ell }}. \end{array}$$

For continuous probability densities defined on the same support, the KL divergence is defined analogously by replacing the discrete sum with an integral. Smaller values indicate that the predicted distribution more closely approximates the observed distribution, whereas larger values indicate greater discrepancy (Kullback and Leibler, 1951). Since KL divergence is defined for probability distributions rather than raw connectivity matrices, we first transformed each functional connectivity matrix into a graph- or topology-derived feature distribution, and then normalized the resulting values to sum to 1. In all KL-based evaluations, the observed distribution was treated as the reference distribution and the predicted distribution as its approximation, so that *D*_KL_(*P*_true_||*Q*_pred_) measures the information lost when the predicted network is used to approximate the true network. Specifically, we computed KL divergence for the following distributions:

(a) **Node strength distribution:** to summarize how weighted connectivity was distributed across brain regions, we examine the node-strength distributions of the predicted and observed functional networks. In a weighted graph, the strength of node *i* is defined as the sum of the weights of all edges incident to that node, that is, $s_{u}=\underset{u}{\sum }w_{uv}$ (Newman, 2018). The corresponding strength distribution describes the fraction of nodes having strength *s*, or equivalently, the probability that a randomly chosen node has strength *s*.
(b) **Motif distributions:** to characterize local connectivity patterns in the predicted and observed functional networks, we analyze motif concentration distributions on thresholded binary graphs. Following the network-motif framework, a motif is defined as a small induced subgraph pattern (Milo et al., 2002; Ahmed et al., 2016; Dey et al., 2019), and we consider the 3-node and 4-node motifs as shown in Figure 5. For each subject, we count the occurrences of each connected motif type separately for 3-node and 4-node subgraphs. The concentration (*C*_δ_) of an all connected *k*-node motif of type δ is the ratio of its number of occurrences (*N*_δ_) to the total number of all connected *k*-node motifs in the network, i.e., $C_{\delta }=\frac{N_{\delta }}{\underset{\delta }{\sum }N_{\delta }}$, where $\underset{\delta }{\sum }N_{\delta }$ is the total number of *k*-node motifs. The resulting motif concentration profile therefore forms a normalized distribution over the set of connected *k*-node motif types.

**Figure 5:**
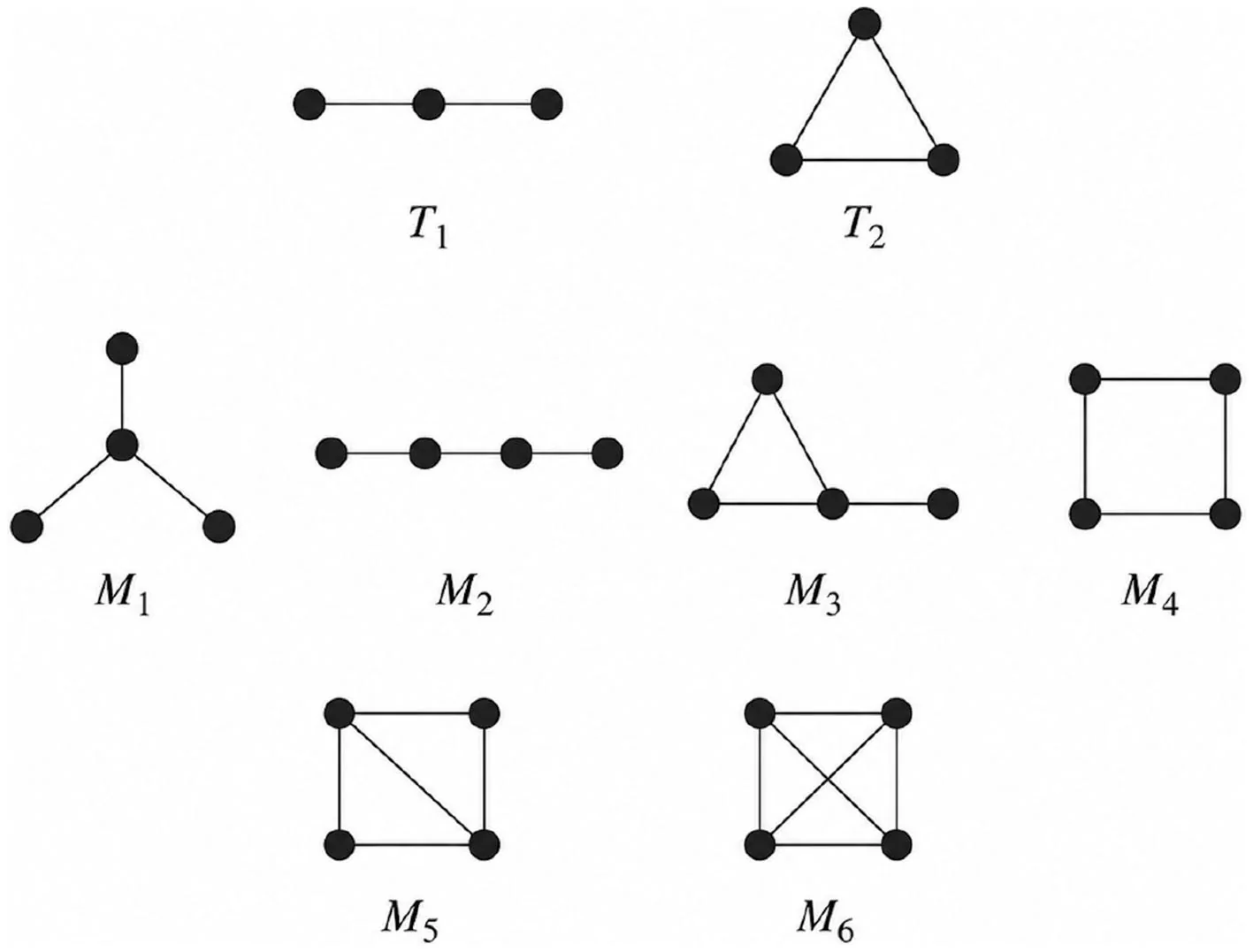
All connected 3-node and 4-node motifs.

### 4.2 Wasserstein distance

Let *D*_1_ and *D*_2_ denote two persistence diagrams. The *r*-Wasserstein distance between them is defined as


$$\begin{array}{l} W_{r}\left(D_{1},D_{2}\right)={\left(\underset{\gamma \in \Gamma \left(D_{1},D_{2}\right)}{\inf}\underset{x\in D_{1}}{\sum }||x-\gamma \left(x\right){||}_{\infty }^{r}\right)}^{1/r}, \end{array}$$

where Γ(*D*_1_, *D*_2_) denotes the set of all matchings between points in the two diagrams, allowing points to be matched to the diagonal when necessary. Smaller values indicate that the two persistence diagrams are more similar, whereas larger values indicate greater topological discrepancy (Ghrist, 2008; Berwald et al., 2018; Panaretos and Zemel, 2019; Dey et al., 2023).

In this study, the Wasserstein distance is computed for persistence diagrams derived from graph filtration. For each predicted functional connectivity matrix and its corresponding observed matrix, persistence diagrams are obtained, and the Wasserstein distance is then used to compare the resulting diagrams. Accordingly, Wasserstein distance served as a direct topological similarity measure between predicted and observed functional connectivity networks, allowing us to assess whether the predicted networks preserved the homology-based organization of the observed brain networks across scales.

## 5 Results

### 5.1 Topological involvement of brain regions

To examine the involvement of individual brain regions in persistent topological structures, we summarize node persistence and node frequency across the 116 brain regions and 998 participants. Figure 6 shows the ten regions with the highest mean values for each measure.

**Figure 6:**
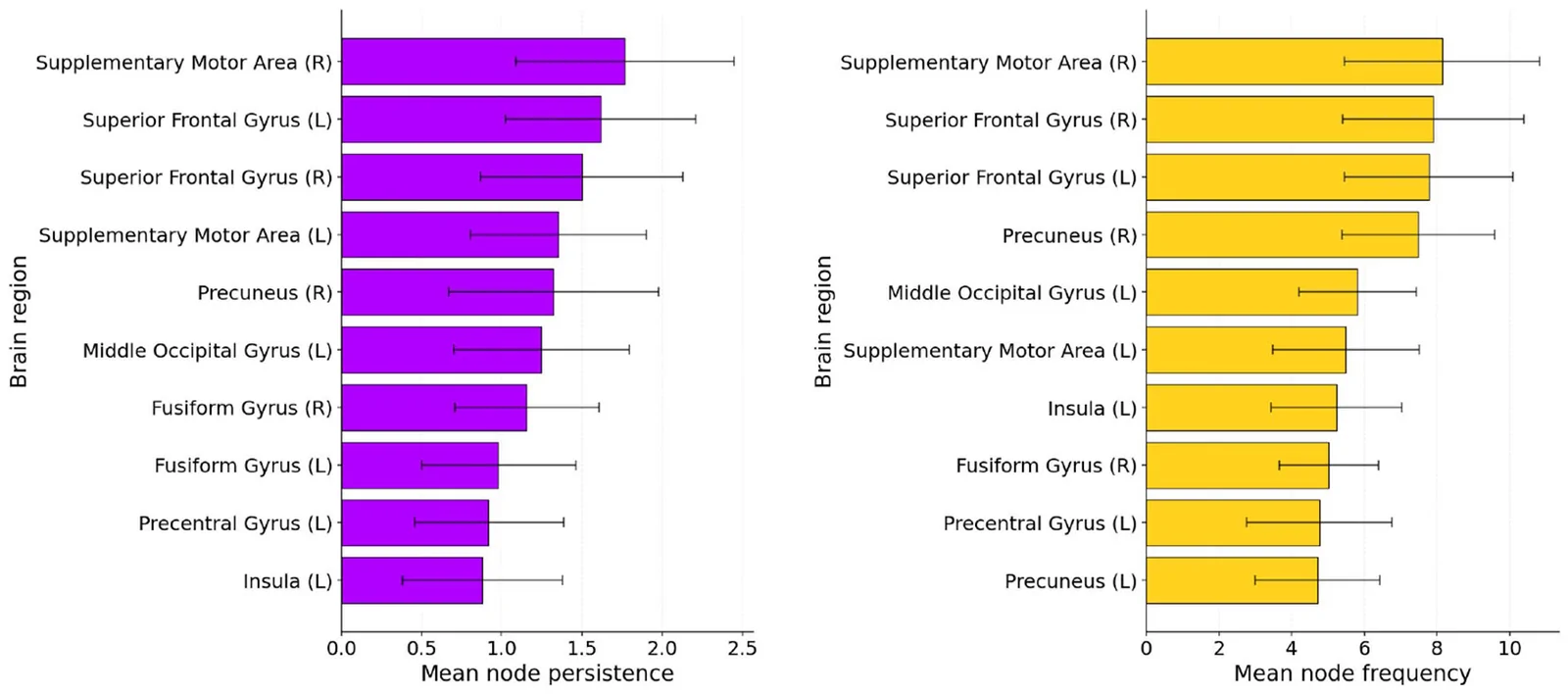
Node persistence and node frequency across brain regions.

The right supplementary motor area shows the largest mean node persistence and node frequency. The bilateral superior frontal gyri and supplementary motor areas also exhibit consistently high values for both measures. Considerable overlap is observed between the two rankings, with nine of the ten highest-ranked regions appearing in both sets. The precuneus, middle occipital gyrus, fusiform gyrus, precentral gyrus, and insula also show strong participation in persistent topological structures.

### 5.2 Visualization and descriptive analysis of TopoGCN prediction

We apply the TopoGCN model to predict subject-specific functional connectivity from corresponding structural connectivity. We evaluate whether the predicted functional connectivities preserve the edge-wise characteristics of the observed functional connectivity matrices. We further assess the extent to which they retain higher-order network organization using geometric and topological performance measures.

A visual comparison of original and predicted functional connectivity for one subject is presented in Figure 7. We find that the predicted functional connectivity matrix closely follows the overall structure of the observed functional connectivity matrix, including its broad block organization and many of the strongest functional connections. The thresholded network visualization further indicates that the predicted connectome preserves much of the dominant connectivity pattern observed in the original network, although some differences remain in the strengths and spatial distribution of individual connections.

**Figure 7:**
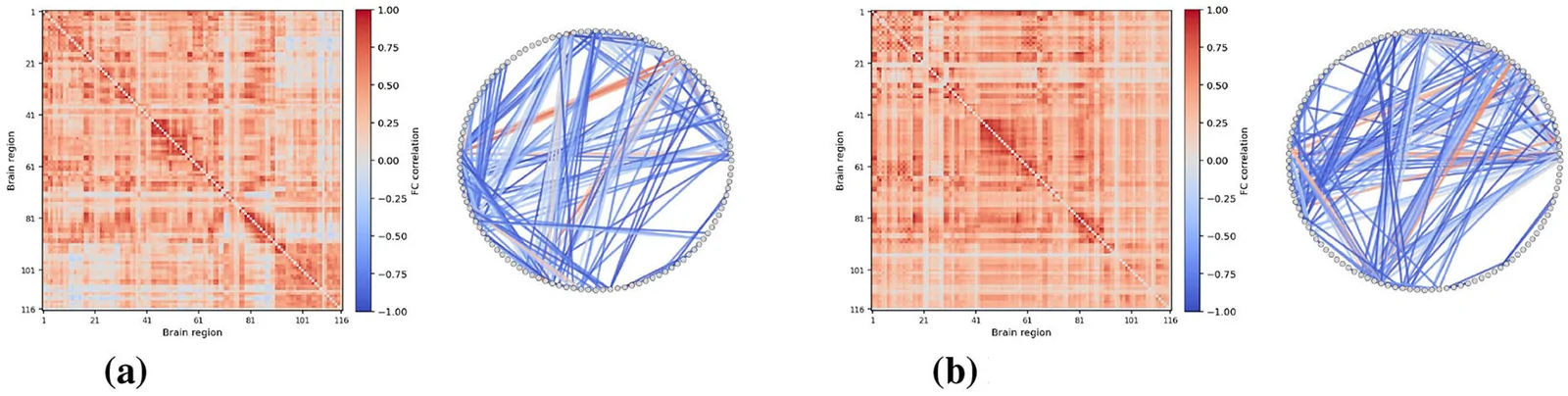
True functional connectivity and predicted functional connectivity using TopoGCN. **(a)** Observed functional connectivity. **(b)** Predicted functional connectivity.

### 5.3 Comparisons among TopoGCN model and other competitors

We evaluate the performance of the proposed TopoGCN model for predicting the functional brain network using the performance metrics defined in Section 4. We compare the TopoGCN model with the following existing state-of-the-art competitors:

- Chen model (Chen et al., 2024): In this model, the structural connectivity matrix serves as a weighted graph, with one-hot region identities used as node features. A two-layer GCN aggregates information from neighboring regions to produce node embeddings, which are then concatenated for each pair of brain regions and passed through a two-layer MLP to predict their functional connectivity.
- Neudorf model (Neudorf et al., 2022): This model uses a Graph Nets architecture in which structural connectivity weights are represented as edge features and nodes are initialized with constant features. The model uses an encode-process-decode graph network architecture with repeated message-passing steps to learn edge-level representations for functional connectivity prediction.
- Li-Mateos model (Li et al., 2022): Li-Mateos model uses a GCN-based graph encoder-decoder architecture to learn the relationship between structural and functional connectivity. A deep GCN encoder integrates structural network information across multiple layers to generate node-level embeddings, with embeddings from intermediate layers concatenated to form the final representation. An outer-product decoder then reconstructs the functional connectivity matrix from these embeddings.
- GCN + Topo: We implement this model using the same GCN encoder and MLP decoder as our proposed TopoGCN framework, while excluding the topological node features. This model evaluates the contribution of topology-aware regularization independently of the topological node features.
- GCN + NP/NF: This model uses the same GCN encoder and MLP decoder as our TopoGCN framework while *excluding* the topology-aware loss. The NP and NF measures are included as node features.

For each evaluation metric, we report the mean matched value, the mean mismatched value, and the corresponding individual-specific effect. Because these metrics quantify dissimilarity between networks, lower matched values indicate greater agreement between the predicted and observed functional connectivity networks. In contrast, a larger individual-specific effect reflects stronger preservation of subject-specific network characteristics.

To assess how well predicted networks preserve connectivity organization, we computed the KL divergence between the node-strength distribution of each observed functional network and that of its predicted counterpart. Node strength is a network measure that summarizes the overall connectivity profile of each brain region. As shown in Table 1, TopoGCN achieves the lowest mean matched KL divergence among all methods while maintaining the largest individual-specific effect. These results indicate that TopoGCN more accurately reproduces the observed node-strength distribution and better preserves subject-specific network organization than competing approaches. The corresponding distributions of subject-level matched and mismatched KL divergences are presented in Supplementary Figure S1. Consistent with the results in Table 1, TopoGCN exhibits the lowest matched KL divergence while also achieving among the lowest mismatched KL divergences across all competing methods.

**Table 1:** KL divergence between the strength distributions of the original and predicted networks.

Model	Mean matched (SE)	Mean mismatched (SE)	Individual-specific effect
Chen	2.511 (0.183)	2.449 (0.050)	−0.062
Neudorf	3.791 (0.231)	3.817 (0.028)	0.026
Li-Mateos	2.706 (0.169)	2.679 (0.038)	−0.027
GCN + Topo	2.302 (0.178)	2.287 (0.043)	−0.015
GCN + NP/NF	2.565 (0.200)	2.600 (0.049)	0.035
TopoGCN	**1.998 (0.157)**	**2.123 (0.037)**	**0.125**

Values are reported as means across subjects, and standard errors are in parentheses. Bold values represent lowest mean matched, lowest mean mismatched, and largest individual-specific effect.

Now, we assess the higher-order network geometry in predicted functional connectivity networks by comparing the distributions (motif concentration) of 3-node and 4-node motifs. Motif concentration profiles capture local connectivity patterns that cannot be characterized by pairwise edges alone and therefore provide an important measure of higher-order network topology.

Tables 2, 3 report the KL divergence between motif concentration distributions derived from the observed and predicted functional connectivity networks. For the 3-node motif analysis (Table 2), TopoGCN achieves the lowest mean matched KL divergence, indicating the closest agreement between predicted and observed motif distributions among all competing methods. We further observe that the individual-specific effects are consistently small and exhibit little variation across methods. Overall, TopoGCN demonstrates the highest fidelity in reproducing the observed 3-node motif concentration profiles, suggesting that it more effectively preserves the local topological organization of functional brain networks. A similar pattern is observed for the 4-node motif analysis (Table 3). TopoGCN again attains the lowest mean matched KL divergence, outperforming all competing approaches and demonstrating the closest agreement with the observed higher-order network structure. Moreover, TopoGCN achieves the largest individual-specific effect, tied with GCN+NP/NF, indicating improved preservation of subject-specific motif organization. The corresponding subject-level matched and mismatched KL-divergence distributions for the 3-node and 4-node motif concentrations are provided in the Supplementary Figures S2, S3. These subject-level distributions exhibit a similar overall pattern as in Tables 2, 3, with TopoGCN attaining lower matched and mismatched KL-divergence values than the competing methods.

**Table 2:** KL divergence between predicted and original 3-node motif concentration distributions.

Model	Mean matched (SE)	Mean mismatched (SE)	Individual-specific effect
Chen	0.020 (0.002)	0.021 (0.001)	0.001
Neudorf	0.104 (0.004)	0.104 (0.003)	0.000
Li-Mateos	0.023 (0.002)	0.023 (0.001)	-0.000
GCN + Topo	0.010 (0.001)	0.011 (0.000)	0.001
GCN + NP/NF	0.015 (0.001)	0.016 (0.001)	**0.001**
TopoGCN	**0.009 (0.001)**	**0.010 (0.000)**	0.000

Values are reported as means across subjects, and standard errors are in parentheses. Bold values represent lowest mean matched, lowest mean mismatched, and largest individual-specific effect.

**Table 3:** KL divergence between predicted and original 4-node motif concentration distributions.

Model	Mean matched (SE)	Mean mismatched (SE)	Individual-specific effect
Chen	0.065 (0.004)	0.066 (0.003)	0.001
Neudorf	0.227 (0.008)	0.227 (0.004)	0.000
Li-Mateos	0.100 (0.005)	0.099 (0.003)	-0.001
GCN + Topo	0.031 (0.002)	0.033 (0.001)	0.001
GCN + NP/NF	0.047 (0.004)	0.049 (0.003)	**0.002**
TopoGCN	**0.030 (0.002)**	**0.032 (0.001)**	**0.002**

Values are reported as means across subjects, and standard errors are in parentheses. Bold values represent lowest mean matched, lowest mean mismatched, and largest individual-specific effect.

Now, we evaluate whether the predicted functional connectivity networks preserve the multiscale topological structure of the observed networks using the Wasserstein distance between persistence diagrams. This analysis consists of two steps. First, we construct persistence diagrams for both the observed functional connectivity network and each predicted network using the graph filtration procedure described in Section 3.1.2. Second, we quantify the dissimilarity between the persistence diagram of the observed network and those of the predicted networks generated by TopoGCN and other competing methods using the 2-Wasserstein distance, *W*_2_. Smaller Wasserstein distances indicate closer agreement in topological structure and therefore better preservation of the underlying higher-order network geometry.

Figure 8 presents the observed functional connectivity matrix, the corresponding predicted matrix using TopoGCN, and their associated persistence diagrams. Persistence diagrams provide a multiscale summary of network topology, with *H*_0_ capturing connected-component structure and *H*_1_ characterizing cycles. Visual inspection indicates that the predicted functional connectivity matrix closely resembles the observed matrix, prevserving major connectivity patterns and network organization. This similarity is further reflected in the persistence diagrams, which exhibit comparable distributions of topological features across both *H*_0_ and *H*_1_. These results suggest that TopoGCN-based prediction effectively captures not only pairwise connectivity relationships but also higher-order topological structures present in the functional brain network.

**Figure 8:**
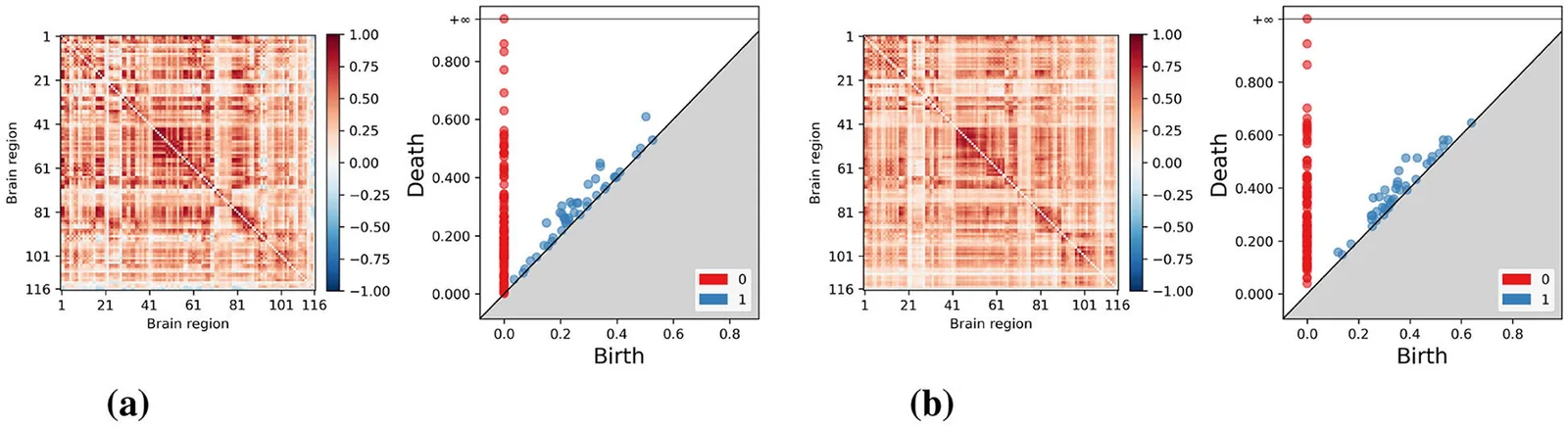
Observed and TopoGCN-based predicted functional connectivity matrices with their corresponding persistence diagrams. **(a)** Observed functional connectivity. **(b)** Predicted functional connectivity.

Table 4 shows that the (mean) Wasserstein distance between the original persistence diagrams and the TopoGCN persistence diagrams is substantially smaller than that between the original persistence diagrams and other competitors. These results suggest that TopoGCN more accurately preserves the multiscale topological organization of functional brain networks. TopoGCN also achieves the largest individual-specific effect, substantially exceeding all competing approaches. This finding indicates that the topological features recovered by TopoGCN are not only closer to the observed networks overall but also better capture subject-specific variations in network topology. The subject-level distributions of matched and mismatched Wasserstein distances between observed and predicted persistence diagrams shown in Supplementary Figure S4 further indicate that TopoGCN tends to produce smaller matched and mismatched values than the competing approaches.

**Table 4:** Wasserstein distance comparison between predicted and observed persistence diagrams.

Model	Mean matched (SE)	Mean mismatched (SE)	Individual-specific effect
Chen	8.079 (0.266)	8.124 (0.183)	0.045
Neudorf	5.454 (0.174)	5.518 (0.035)	0.064
Li-Mateos	11.382 (0.345)	11.340 (0.263)	-0.043
GCN + Topo	5.263 (0.182)	5.301 (0.098)	0.038
GCN + NP/NF	9.140 (0.277)	9.179 (0.181)	0.040
TopoGCN	**4.950 (0.176)**	**5.058 (0.092)**	**0.108**

Values are reported as means across subjects, and standard errors are in parentheses. Bold values represent lowest mean matched, lowest mean mismatched, and largest individual-specific effect.

Collectively, these findings indicate that TopoGCN more accurately reconstructs the topological organization of functional brain networks across multiple scales. In addition to preserving edge-wise connectivity patterns, TopoGCN maintains higher-order motif structure and persistence-based topological features, resulting in improved recovery of the intrinsic geometry of functional connectomes.

### 5.4 Hypothesis tests for comparing Betti curves

In this section, we compare the Betti curves of the observed and TopoGCN-predicted functional connectivity networks using the Kolmogorov–Smirnov test (Kolmogorov, 1933; Smirnov, 1948; Conover, 1999) and the Anderson–Darling test (Anderson and Darling, 1952, 1954). Figure 9 compares the Betti curves (defined in Section 3.1.2) derived from the observed and predicted functional connectivity networks across the entire filtration range. The predicted functional connectivity networks exhibit topological evolution that closely matches that of the observed networks. In particular, the β_0_ curves exhibit highly similar decreasing trajectories, indicating that the predicted networks accurately capture the progressive merging of connected components as the filtration threshold increases. Likewise, the β_1_ curves exhibit similar growth patterns, suggesting that the model successfully captures the formation and accumulation of higher-order cyclic structures throughout the filtration process.

**Figure 9:**
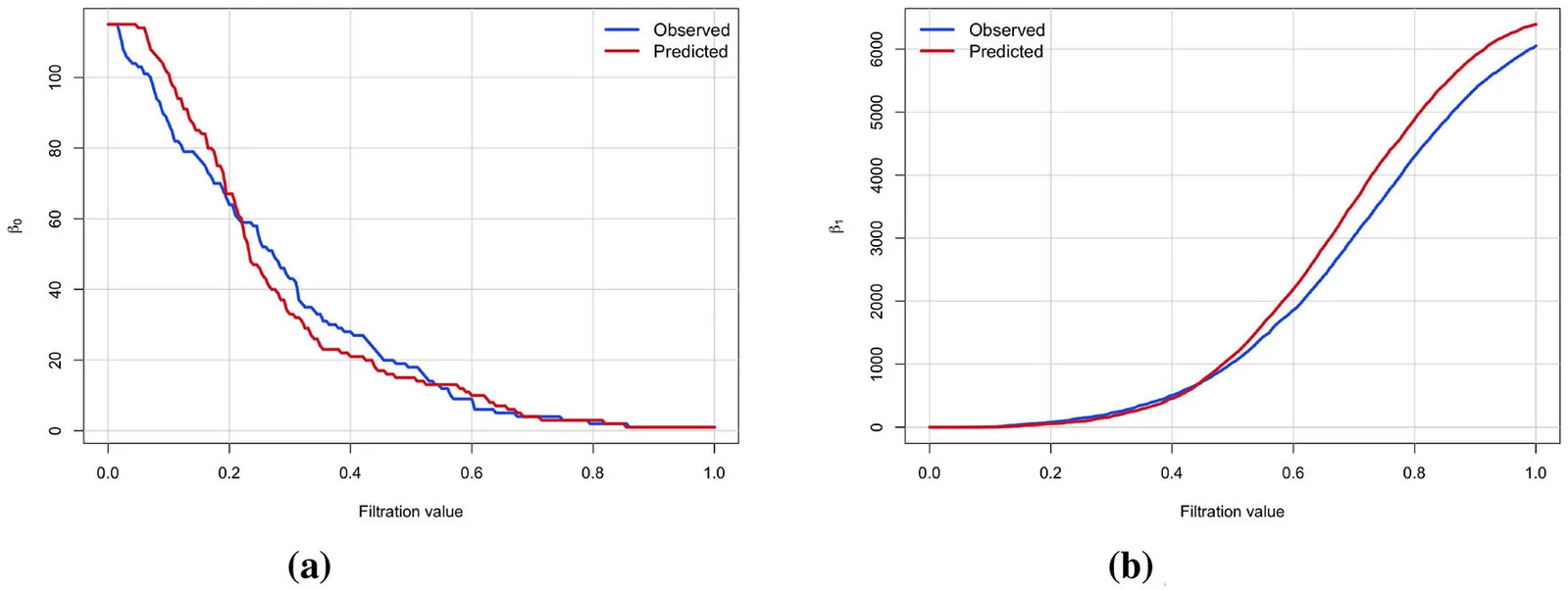
Comparison of the β_0_ and β_1_ curves of the observed and TopoGCN-predicted functional connectivity networks for one subject. **(a)** β_0_ curve. **(b)** β_1_ curve.

The quantitative results in Table 5 further support these observations. For both β_0_ and β_1_ curves, the Kolmogorov–Smirnov and Anderson–Darling tests yield *p*-values greater than 0.10, indicating no statistically detectable differences between the distributions of the observed and predicted Betti curves. These findings provide strong evidence that the predicted functional connectivity networks using TopoGCN preserve both connected-component and cycle-based topological characteristics, thereby retaining key higher-order organizational properties of the observed functional connectome.

**Table 5:** Kolmogorov–Smirnov and Anderson–Darling test results for β_0_ and β_1_ curves.

	β_0_ Curve		β_1_ Curve	
Test	Test statistic	*p*-value	Test statistic	*p*-value
Kolmogorov–Smirnov	0.0846	0.4685	0.0796	0.5474
Anderson–Darling	0.8208	0.4665	1.7282	0.1297

We also compute subject-level *p*-values for each test subject by comparing the observed and predicted Betti curves. Figure 10 summarizes the distributions of the resulting Kolmogorov–Smirnov (KS) and Anderson–Darling (AD) test *p*-values across all test subjects using boxplots. The KS test yields *p*-values exceeding the conventional significance threshold of 0.05 for most test subjects for both β_0_ and β_1_, indicating no statistically significant difference between the observed and predicted Betti curves in most cases. The AD test produces generally smaller *p*-values than the KS test, reflecting its greater sensitivity to discrepancies in the tails of the distributions. Nevertheless, for most of the subjects, the AD *p*-values also remain above 0.05, providing further evidence that the predicted Betti curves closely match the observed topological summaries.

**Figure 10:**
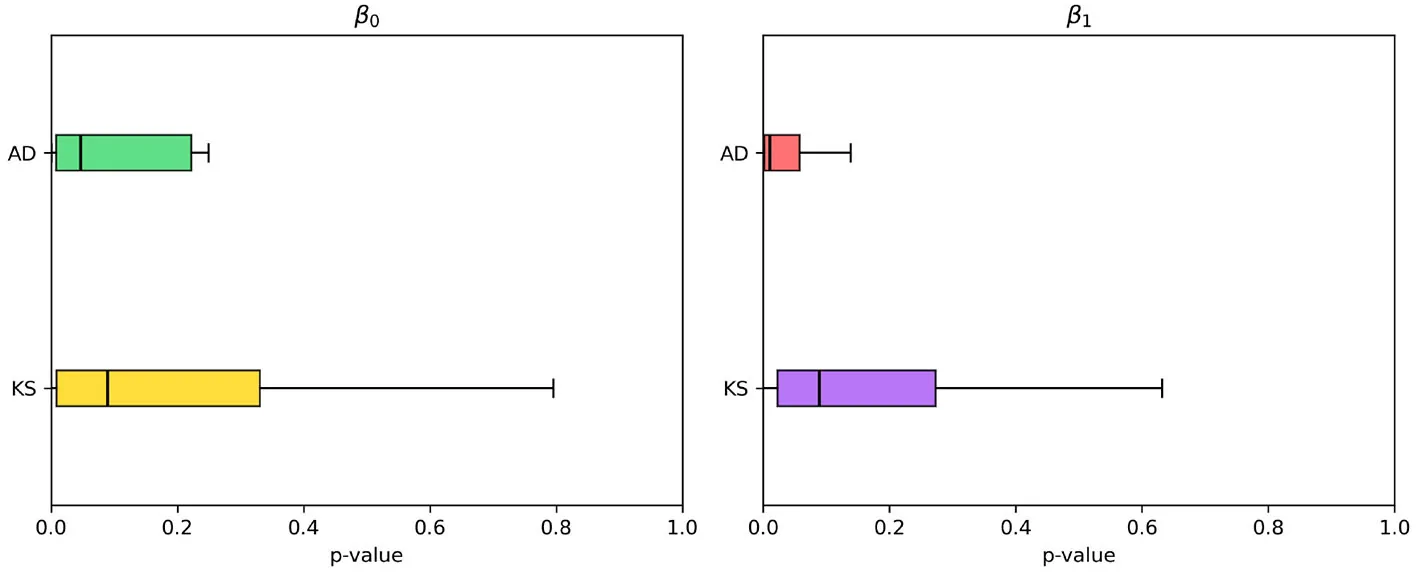
Distribution of *p*-values obtained from Kolmogorov–Smirnov (KS) and Anderson–Darling (AD) tests comparing the observed and predicted β_0_ and β_1_ curves. We conducted an additional experiment by applying TopoGCN to a small schizophrenia connectome dataset (Vohryzek et al., 2020). The results are presented in Supplementary Section 2.

## 6 Discussion

In this study, we introduced TopoGCN, a topology-informed graph convolutional network framework for predicting subject-specific functional connectivity from structural brain connectivity. Unlike existing structure-function prediction models that primarily focus on edge-wise reconstruction, TopoGCN integrates topological data analysis into both the feature-learning and optimization stages of the model. Specifically, persistent homology-derived node features capture each brain region's participation in higher-order cycle structures, while a topology-aware loss function encourages preservation of multiscale topological organization in the predicted functional connectomes. In addition to existing TDA-based approaches for brain connectivity analysis, TopoGCN contributes by incorporating topological information directly into the structure-to-function prediction process. Specifically, persistent homology is used not only to characterize connectivity patterns but also to inform node representations and model optimization through a topology-aware loss. This integration allows TopoGCN to account for multiscale topological characteristics when predicting functional connectivity, complementing conventional graph-based representations.

Using structural and functional connectome data from the Human Connectome Project, we demonstrated that TopoGCN consistently outperforms several state-of-the-art graph-based approaches in preserving network-level and topological characteristics of functional brain organization. The proposed framework achieved lower divergences in node-strength and motif distributions, smaller Wasserstein distances between persistence diagrams, and stronger preservation of topological signatures. Furthermore, the close agreement between observed and predicted Betti curves indicates that TopoGCN successfully captures the evolution of both connected-component and cycle structures across filtration scales. These findings suggest that higher-order topological information provides complementary information beyond conventional graph representations and substantially improves the modeling of structure-function coupling.

Future work may extend this framework to dynamic functional connectivity, disease-specific cohorts, and longitudinal neuroimaging studies, as well as explore richer topological descriptors and attention-based graph architectures. These directions may contribute to a deeper understanding of how anatomical structure gives rise to functional brain dynamics and provide new tools for precision neuroscience and biomarker discovery.

## Data Availability

Publicly available datasets were analyzed in this study. This data can be found here: All data is made publicly available by the Human Connectome Project (Van Essen et al., 2013).
