# Supplementary material for: Enhancing structural to functional brain network prediction using topological graph learning
Source: Front Neurosci. 2026 Sep 28;20:1939985. doi: 10.3389/fnins.2026.1939985 (PMC13655150; doi:10.3389/fnins.2026.1939985)
Supplement: Supplementary file 1 [file Data_Sheet_1.pdf]

Supplementary Material: Enhancing Structural to Functional Brain Network Prediction Using Topological Graph Learning

1 SUPPLEMENTARY RESULTS FOR HUMAN CONNECTOME DATASET

1.1 Model training and implementation details

To assess the sensitivity of the proposed model to the weighting of the topology-aware loss, we evaluated λ_{topo} over the values 0.10, 0.20, 0.50, and 0.75, while keeping the remaining model architecture unchanged. The corresponding prediction performance is summarized in Table S1.

Table S1. Prediction performance and selection of λ_{topo}.

λ_{topo}	MSE	MAE	Mean correlation
**0.10**	**0.030**	**0.137**	**0.713**
0.20	0.031	0.139	0.696
0.50	0.033	0.144	0.668
0.75	0.035	0.147	0.654

The results indicate that prediction performance gradually decreases as λ_{topo} increases beyond 0.10. In particular, larger values of λ_{topo} lead to increasing MSE and MAE and decreasing mean subject-level correlation. These findings suggest that excessive weighting of the topology-aware objective can reduce edge-level connectivity prediction performance.

1.2 Node strength distribution

The Figure S1 shows that TopoGCN has the lowest matched KL divergences and also the lowest mismatched KL divergences among the compared methods. In particular, the matched distribution for TopoGCN is shifted lower than those of other methods, indicating closer agreement between the predicted and empirical node-strength distributions. At the same time, the gap between the matched and mismatched distributions remains visible for TopoGCN, which suggests that its predictions are not only accurate on average but also better aligned with the correct subject than with other subjects.

1.3 Motif distribution

This Figure S2 shows that TopoGCN and GCN + Topo have the lowest matched KL divergences among the compared methods, indicating the closest agreement with the empirical 3-node motif concentration distributions. In contrast, Neudorf shows substantially larger matched and mismatched divergences, reflecting weaker preservation of local motif structure. The mismatched distributions remain slightly higher than the matched distributions for TopoGCN, which suggests that its predicted motif profiles align better with the correct subject than with other subjects.

The 4-node motif analysis leads to a similar conclusion. TopoGCN and GCN + Topo achieve the lowest matched and mismatched KL divergences, indicating the closest agreement with the empirical 4-node motif concentration distributions, whereas Neudorf and Li-Mateos exhibit substantially larger divergences.

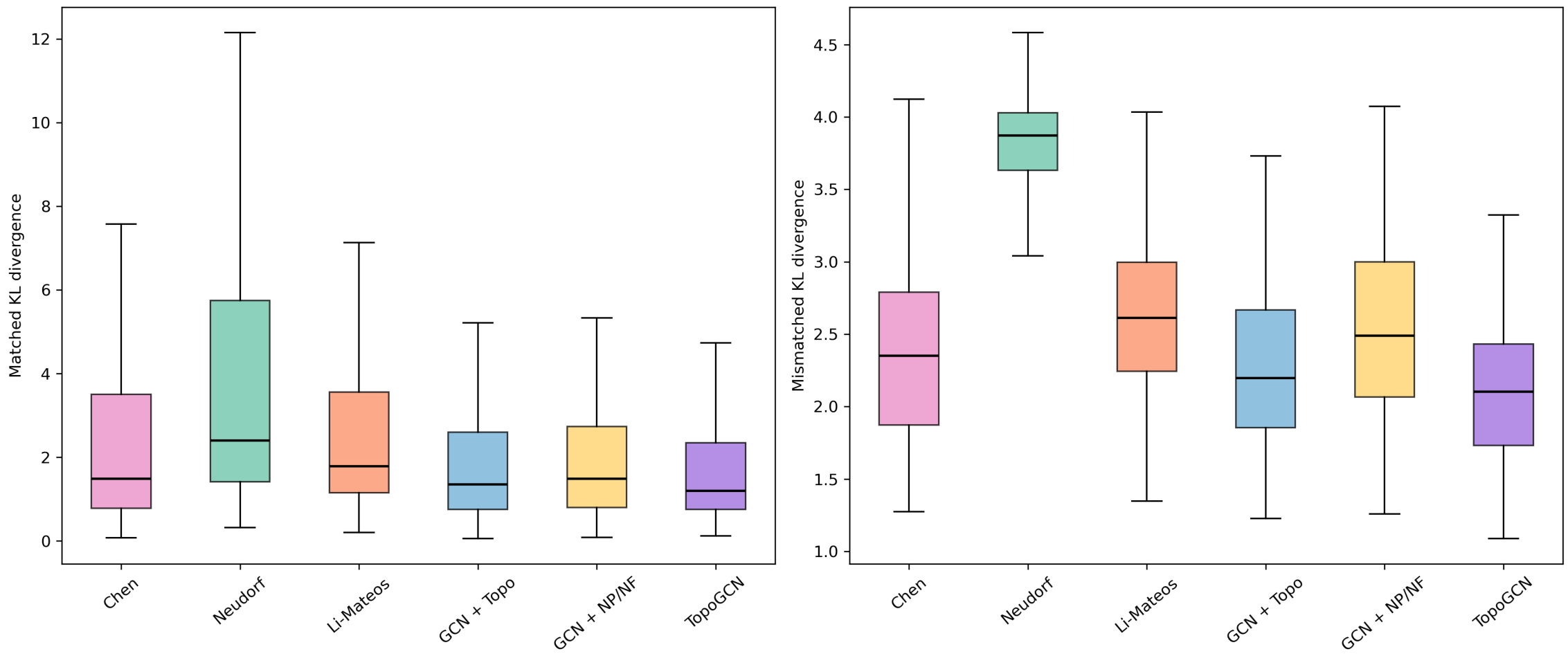

Figure S1. KL divergence between the strength distributions of the original and predicted networks.

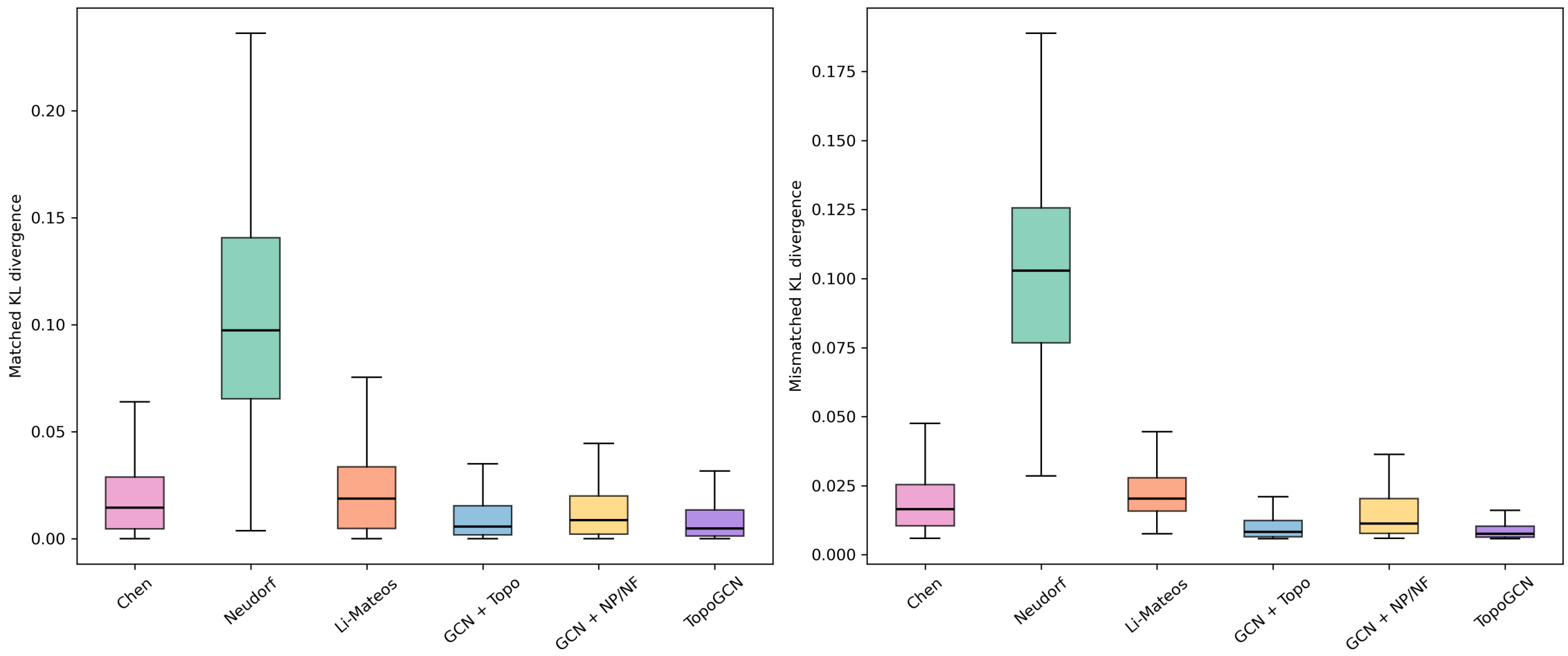

Figure S2. KL divergence between predicted and original 3-node motif concentration distributions.

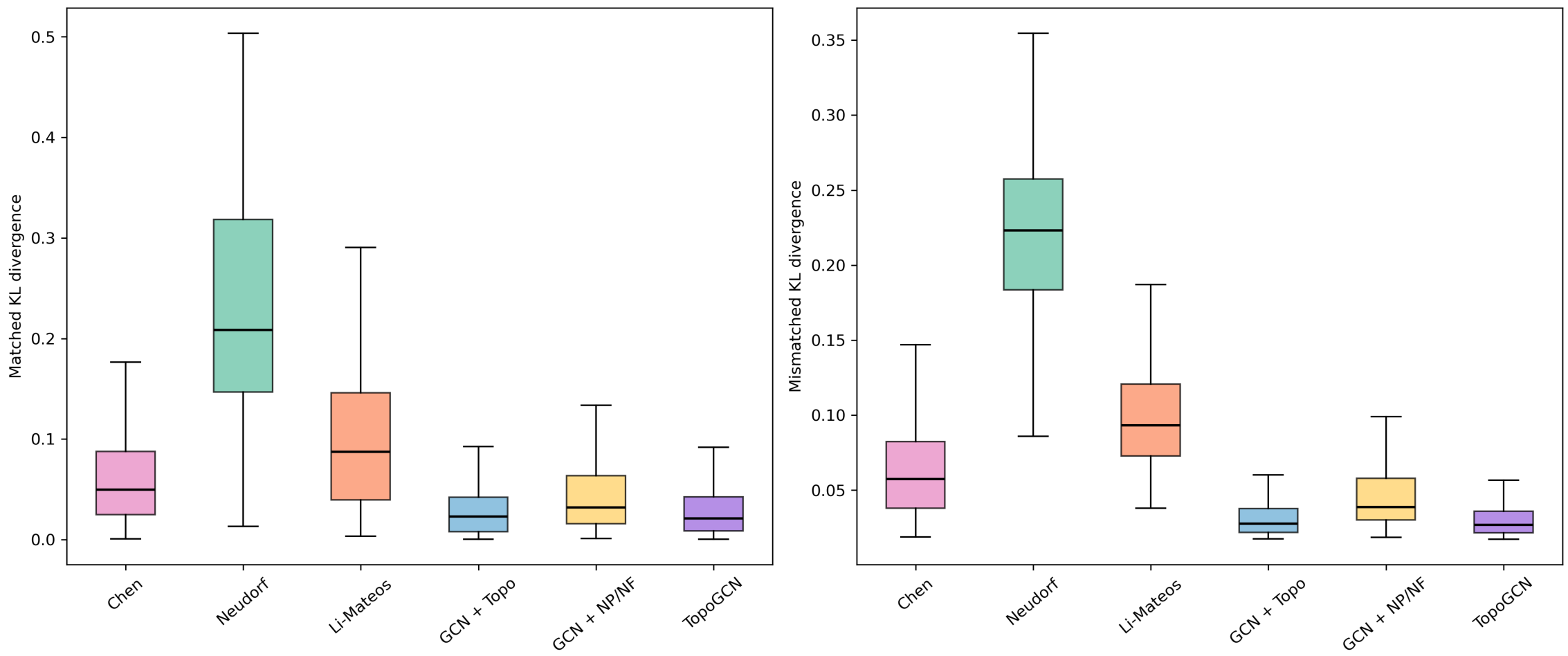

Figure S3. KL divergence between predicted and original 4-node motif concentration distributions.

1.4 Wasserstein Distance

Figure S4 indicates that TopoGCN attains the lowest matched and mismatched Wasserstein distances among the compared methods, whereas Li-Mateos and GCN + NP/NF yield noticeably larger distances. This suggests that the persistence diagrams derived from the functional connectivity networks predicted by TopoGCN remain closer to the observed persistence diagrams than those derived from the competing approaches.

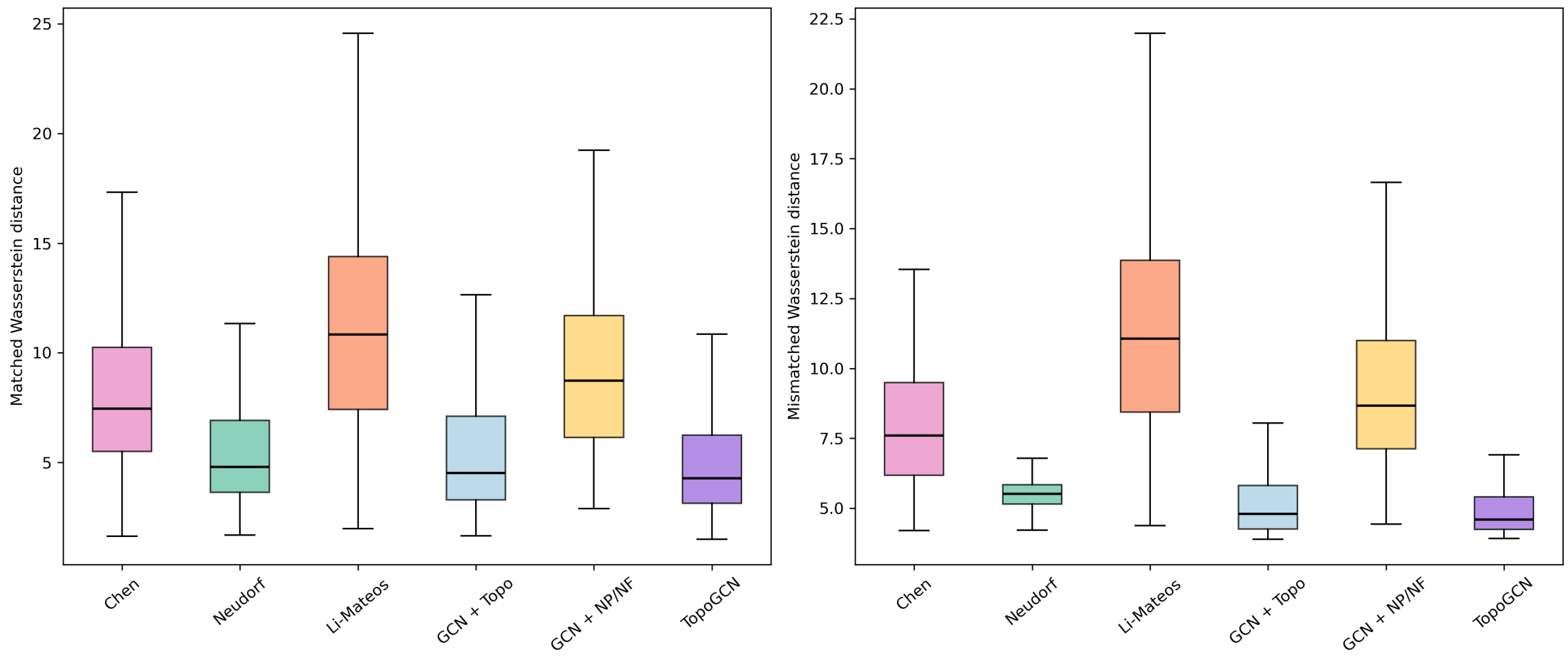

Figure S4. Wasserstein distance comparison between predicted and observed persistence diagrams.

2 SUPPLEMENTARY RESULTS FOR SCHIZOPHRENIC DATASET

To examine the applicability of TopoGCN to a disease-specific cohort, we additionally consider a publicly available schizophrenia connectome dataset (Vohryzek et al., 2020) containing paired structural and functional connectivity matrices. The dataset includes 27 individuals with schizophrenia and provides connectivity matrices at multiple Lausanne parcellation resolutions (Cammoun et al., 2012). In the present analysis, we use the 129-region parcellation.

Given the limited sample size of the schizophrenia cohort, all 27 participants are used for model fitting without splitting the data into separate training, validation, and test sets. For each participant, node persistence and node frequency are computed from the structural connectivity network and combined with node-identity features as inputs to TopoGCN. We then apply the TopoGCN implementation described in Section 3.2.3 of the main manuscript to the schizophrenia cohort, with the input dimension adjusted to accommodate the 129-region parcellation.

Figure S5 shows the observed and predicted functional connectivity matrices. We find that the predicted functional connectivity matrices capture the overall connectivity structure observed in the empirical matrices, although some differences remain in the strength and distribution of individual connections. Given the limited sample size and the use of all 27 participants for model fitting, the results primarily illustrate the feasibility of applying TopoGCN to schizophrenia connectomes, while a larger independent clinical cohort is needed for a more extensive evaluation of generalizability.

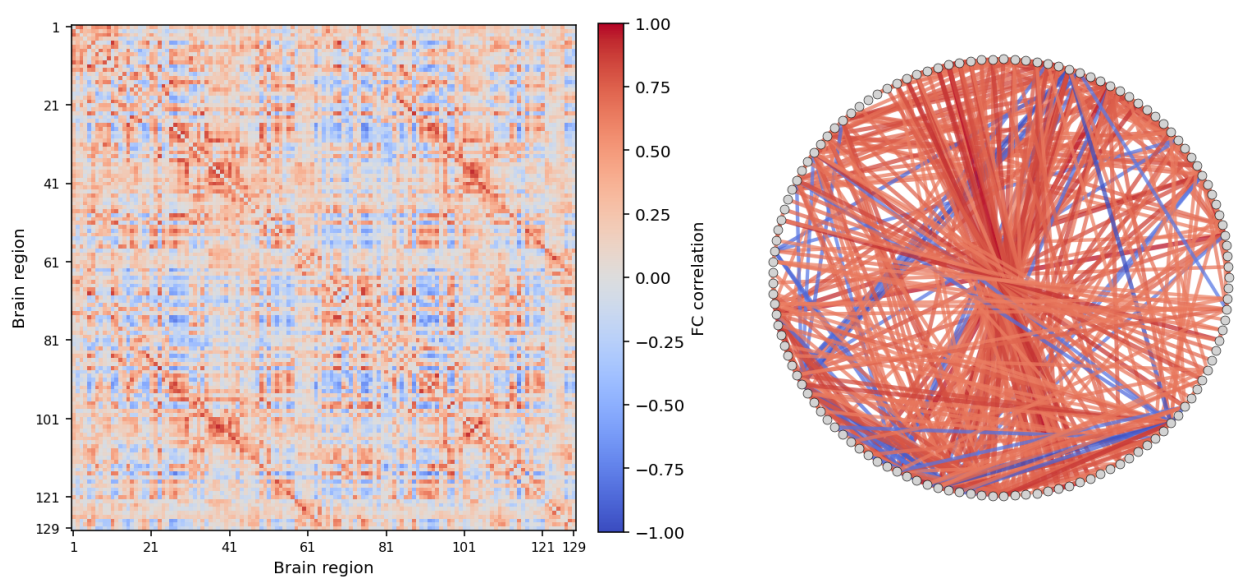

(a) Observed functional connectivity.

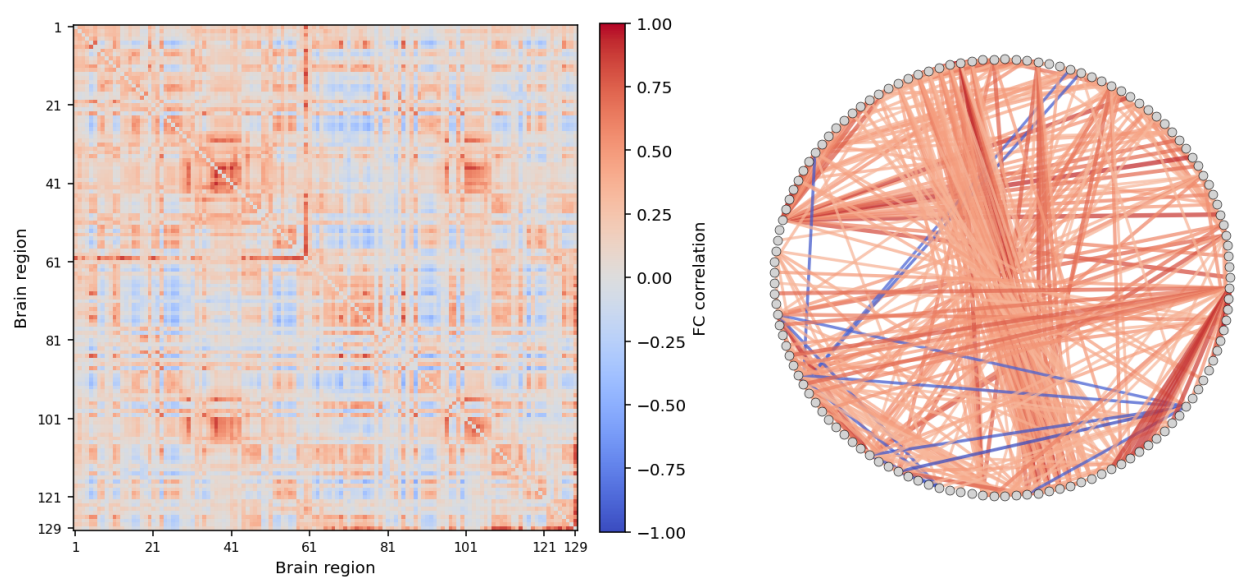

(b) Predicted functional connectivity.

Figure S5. True functional connectivity and predicted functional connectivity using TopoGCN for the schizophrenia connectome dataset.

REFERENCES

Cammoun, L., Gigandet, X., Meskaldji, D., Thiran, J. P., Sporns, O., Do, K. Q., et al. (2012). Mapping the human connectome at multiple scales with diffusion spectrum mri. *Journal of Neuroscience Methods* 203, 386–397. doi:https://doi.org/10.1016/j.jneumeth.2011.09.031

[Dataset] Vohryzek, J., Aleman-Gomez, Y., Griffa, A., Raoul, J., Cleusix, M., Baumann, P. S., et al. (2020). Structural and functional connectomes from 27 schizophrenic patients and 27 matched healthy adults. doi:10.5281/zenodo.3758534
